# Supplementary material for: Single-cell transcriptomic profiling unveils dysregulation of cardiac progenitor cells and cardiomyocytes in a mouse model of maternal hyperglycemia
Source: Commun Biol. 2022 Aug 15;5:820. doi: 10.1038/s42003-022-03779-x (PMC9378651; doi:10.1038/s42003-022-03779-x)
Supplement: Supplementary file 2 — Supplementary Information [file 42003_2022_3779_MOESM2_ESM.pdf]

Supplementary Information

Single-cell Transcriptomic Profiling Unveils Dysregulation of Cardiac Progenitor Cells and Cardiomyocytes in a Mouse Model of Maternal Hyperglycemia

Authors: Sathiyanarayanan Manivannan[1,2#], Corrin Mansfield[1,2#], Xinmin Zhang[3], Karthik. M. Kodigepalli[4], Uddalak Majumdar[1,2], Vidu Garg[1,2,5,6], and Madhumita Basu[1,2,5*]

Affiliations:

[1] Center for Cardiovascular Research, Abigail Wexner Research Institute at Nationwide Children's Hospital, Columbus, Ohio, United States of America. [2] Heart Center, Nationwide Children's Hospital, Columbus, Ohio, United States of America. [3] BioInfoRx Inc. Madison, Wisconsin, United States of America. [4] Department of Pediatrics, Medical College of Wisconsin, Milwaukee, Wisconsin, United States of America. [5] Department of Pediatrics, The Ohio State University College of Medicine, Columbus, Ohio, United States of America.[6] Department of Molecular Genetics, The Ohio State University, Columbus, Ohio, United States of America.

Supplementary Figures and Captions

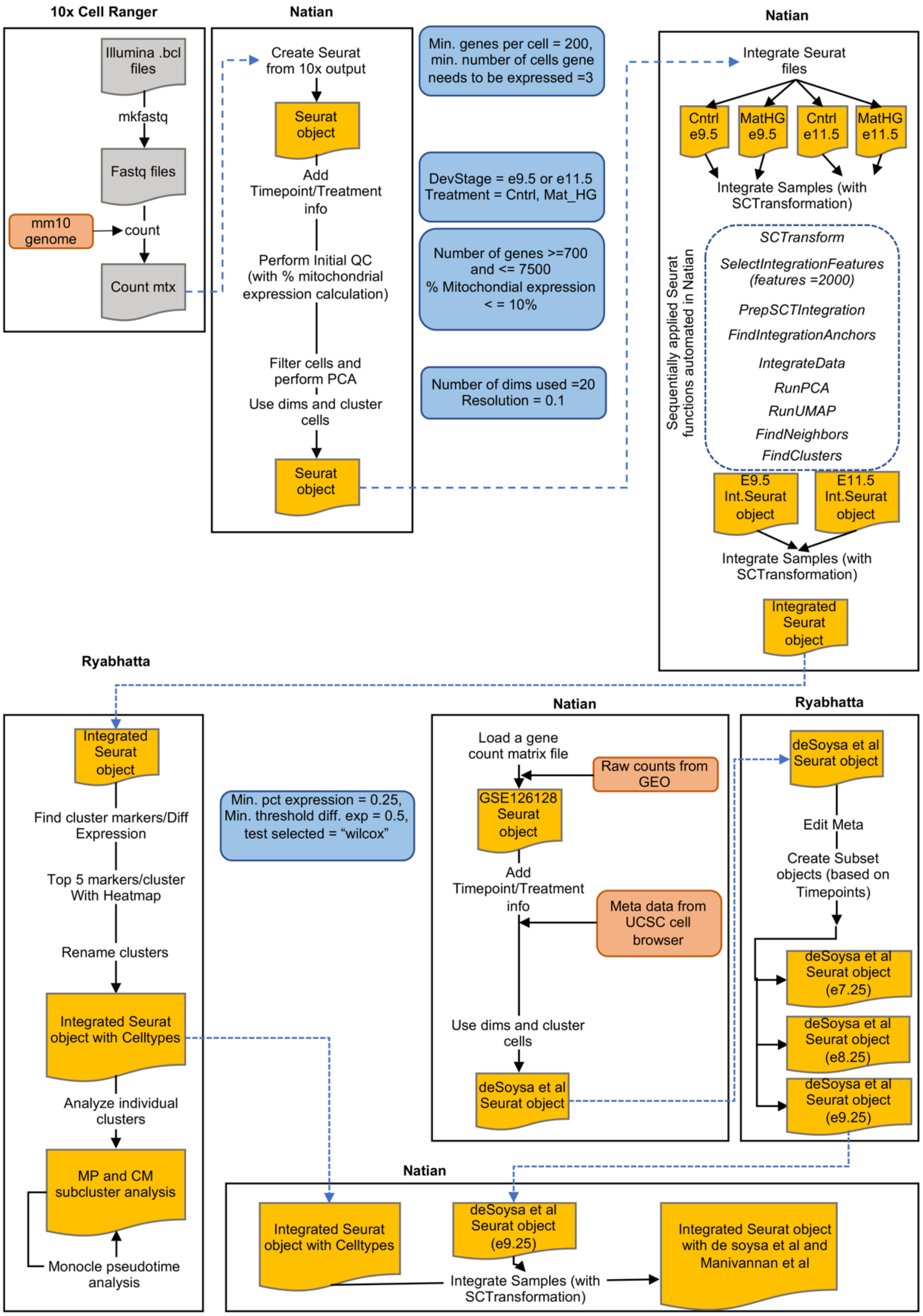

Supplementary Figure 1: Data processing pipelines and filtering cutoffs

Flow diagram shows processing pipelines used to process and integrate individual samples. Primary processing steps were done using 10x Genomics Cell Ranger. The secondary processing and integration of individual samples were performed using Natian and Ryabhatta (Graphical user interfaces to run Seurat processing steps and analysis functions). Input data (references, GEO counts, metadata) to each step shown in salmon box, individual primary processing files shown in grey document shapes, secondary process files shown in orange document shapes. Parameters used for filtering and processing steps shown in blue boxes. Seurat functions used in integration in Natian (implicitly run) are shown in box with dotted outline. Data moved from one app to another are shown in blue dotted lines. A free (open access) manual is available through the following link: https://natian-and-ryabhatta.web.app/.

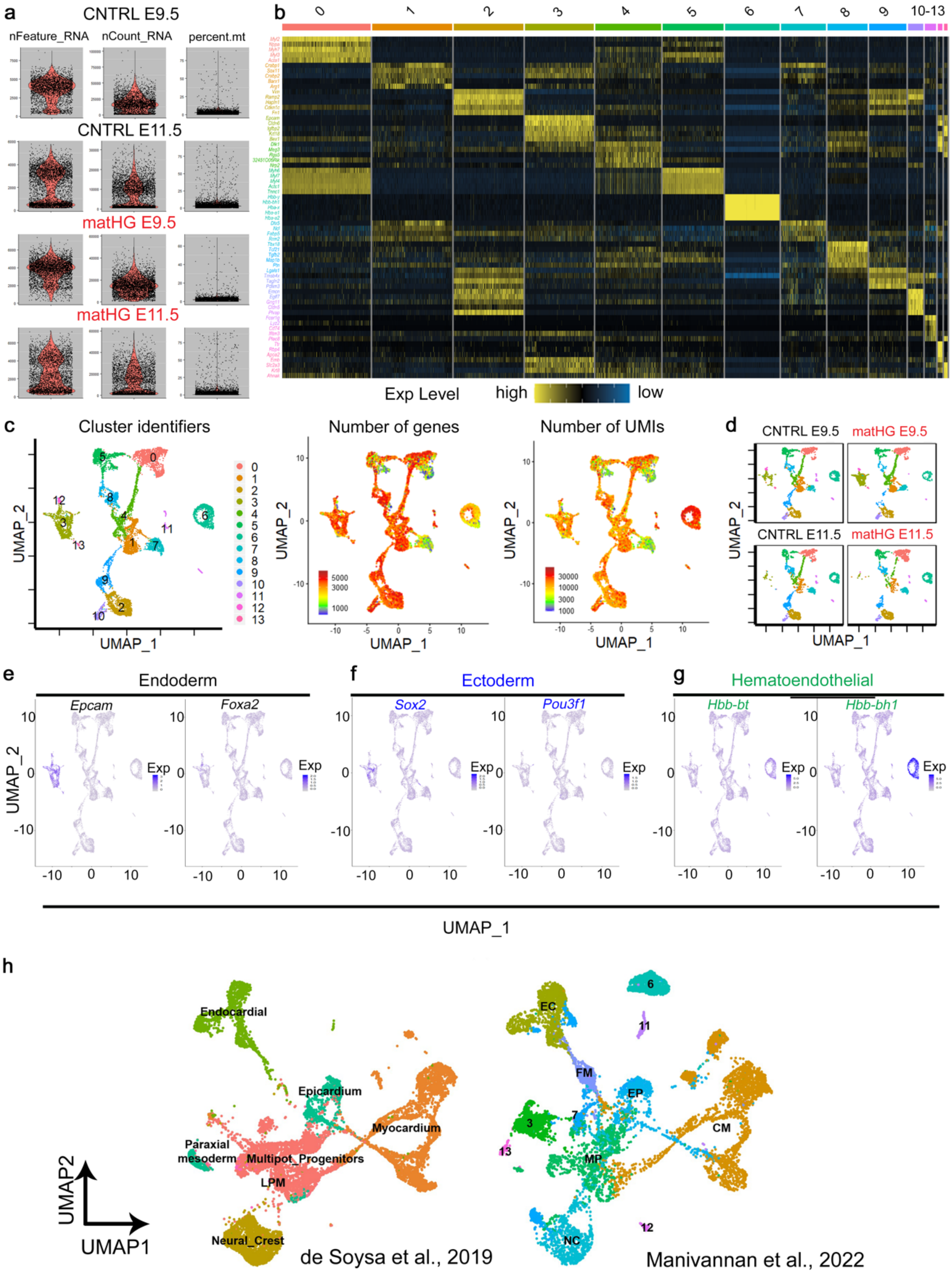
a
CNTRL E9.5
nFeature_RNA
nCount_RNA
percent.mt
CNTRL E11.5
matHG E9.5
matHG E11.5
b
10-13
Exp Level
high
low
c
Cluster identifiers
Number of genes
Number of UMIs
UMAP_2
UMAP_1
d
CNTRL E9.5
matHG E9.5
CNTRL E11.5
matHG E11.5
e
Endoderm
Epcam
Foxa2
f
Ectoderm
Sox2
Pou3f1
g
Hematoendothelial
Hbb-bt
Hbb-bh1
Exp
h
Endocardial
Epicardium
Myocardium
Paraxial mesoderm
Multipot_Progenitors
LPM
Neural_Crest
de Soysa et al., 2019
EC
FM
EP
CM
MP
NC
Manivannan et al., 2022
UMAP2
UMAP1

Supplementary Figure 2. Quality control and normalization of *in vivo* scRNA-seq data

(a) Quality control metrices used to assess the quality of 10xscRNA-seq libraries from CNTRL and matHG-exposed E9.5 and E11.5 hearts. Violin plots illustrating number of genes detected in each cell (nFeature-RNA), unique molecular identifiers (nCount-RNA) and less than 10% of the reads mapped to mitochondrial genes in each of the four cardiac tissue samples. **(b)** Unsupervised clustering shows a total of 14 clusters (0-13) and the top five marker genes per cluster in the heatmap. Normalized log expression levels are shown in yellow (high expression) and dark blue (low expression). **(c, d)** UMAP plots show the cluster identities and relationship between the number of genes and UMIs in merged datasets and in all four samples. **(e-g)** UMAP plots showing the expression of *Epcam*$^{+}$, *Foxa2*$^{+}$ endodermal, *Sox2*$^{+}$, *Pou3f1*$^{+}$ ectodermal and *Hbb-bt*$^{+}$*Hbb-bh1*$^{+}$ hematoendothelial clusters were discarded from subsequent analysis**. (h)** UMAP-plot (left panel) shows single-cell data from de Soysa et al., 2019, E9.25 *wt* embryonic heart cells (GSE126128) representing cell types labelled per metadata information from authors[1]. UMAP-plot (right panel) shows single-cell data from current study, showing labels identified using marker genes in different clusters. UMAP plots generated using Ryabhatta from integrated data with both deSoysa et al;[1] and data from our study (see **Supplementary Fig. 1** for data processing details). CNTRL, control, matHG, maternal hyperglycemia, wt, wildtype, UMAP, Uniform manifold approximation and projection, UMI, unique molecular identifiers, MP, multipotent progenitors, EP, epicardial, NC, neural crest, EC, endocardial/endothelial, FM, fibromesenchymal, CM, cardiomyocytes, LPM, lateral plate mesoderm.

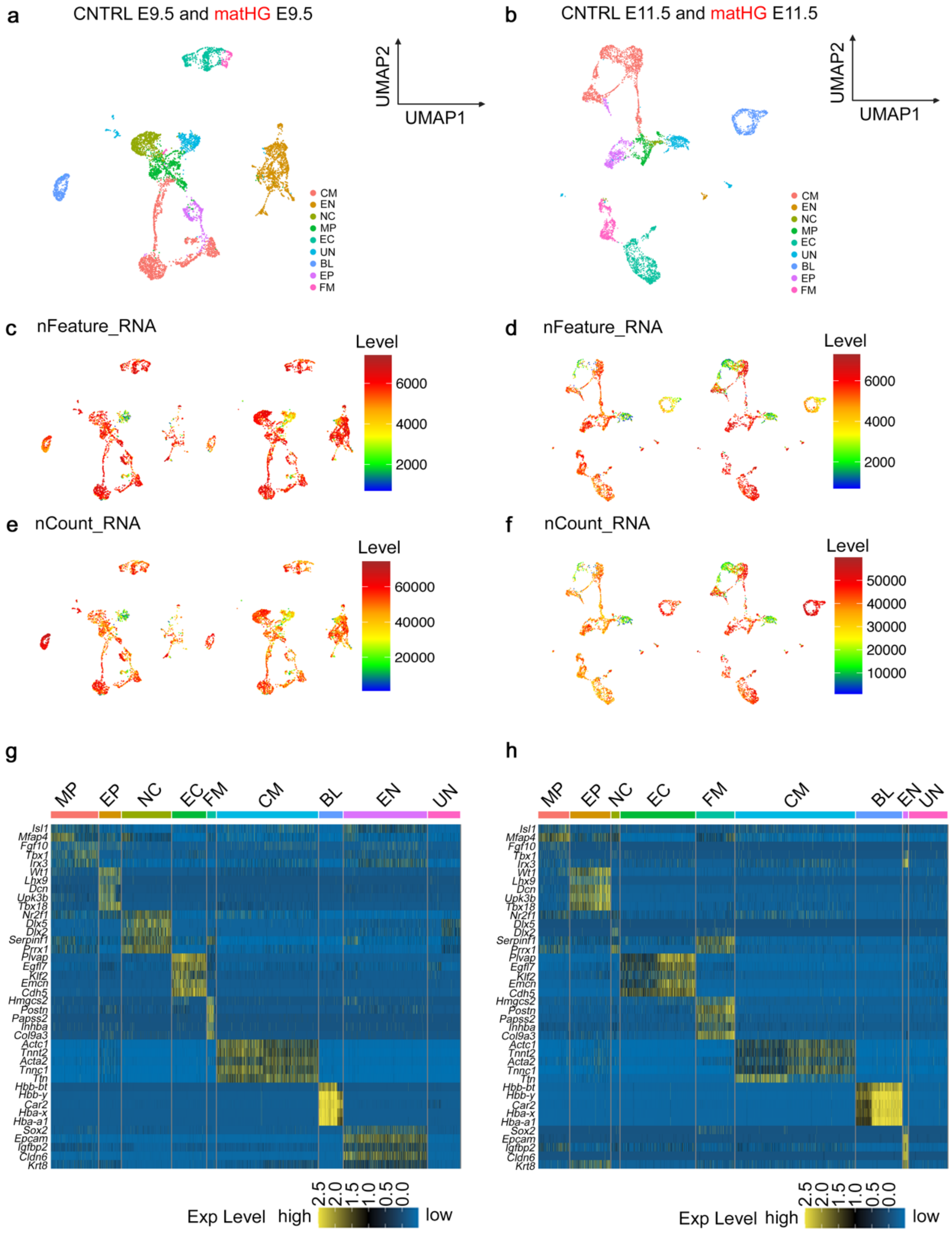
a
CNTRL E9.5 and matHG E9.5
UMAP2
UMAP1
CM
EN
NC
MP
EC
UN
BL
EP
FM
b
CNTRL E11.5 and matHG E11.5
UMAP2
UMAP1
CM
EN
NC
MP
EC
UN
BL
EP
FM
c
nFeature_RNA
Level
6000
4000
2000
d
nFeature_RNA
Level
6000
4000
2000
e
nCount_RNA
Level
60000
40000
20000
f
nCount_RNA
Level
50000
40000
30000
20000
10000
g
MP EP NC EC FM CM BL EN UN
Isl1 Mfap4 Fgf10 Tbx1 Irx3 Wt1 Lhx9 Dcn Upk3b Tbx18 Nr2f1 Dlx5 Dlx2 Serpinf1 Prrx1 Plvap Egfl7 Klf2 Emcn Cdh5 Hmgcs2 Postn Papss2 Inhba Col9a3 Actc1 Tnnt2 Acta2 Tnnc1 Ttn Hbb-bt Hbb-y Car2 Hba-x Hba-a1 Sox2 Epcam Igfbp2 Cldn6 Krt8
2.5 2.0 1.5 1.0 0.5 0.0
Exp Level high
low
h
MP EP NC EC FM CM BL EN UN
Isl1 Mfap4 Fgf10 Tbx1 Irx3 Wt1 Lhx9 Dcn Upk3b Tbx18 Nr2f1 Dlx5 Dlx2 Serpinf1 Prrx1 Plvap Egfl7 Klf2 Emcn Cdh5 Hmgcs2 Postn Papss2 Inhba Col9a3 Actc1 Tnnt2 Acta2 Tnnc1 Ttn Hbb-bt Hbb-y Car2 Hba-x Hba-a1 Sox2 Epcam Igfbp2 Cldn6 Krt8
2.5 2.0 1.5 1.0 0.5 0.0
Exp Level high
low

Supplementary Figure 3. Developmental stage specific unsupervised clustering of control and matHG-exposed scRNA-seq data

(a, b) UMAP plots show distribution of nine clusters (CM, EN, NC, MP, EC, UN, BL, EP, FM) separated based on developmental stages (E9.5 and E11.5), when exposed to maternal CNTRL and HG environment. Colors indicate cluster identities. **(c-f)** Individual UMAP plots show the relationship between the number of genes (nFeature-RNA) and UMIs (nCount-RNA) in E9.5 and E11.5 samples subjected to intrauterine CNTRL and matHG environment. **(g, h)** Unsupervised clustering of CNTRL and matHG-exposed E9.5 and E11.5 single cell transcriptomes show nine clusters and the top five marker genes per cluster in the heatmap. Normalized log expression levels are shown in yellow (high expression) and dark blue (low expression). CNTRL, control, matHG, maternal hyperglycemia, CM, cardiomyocytes, EN, endoderm, NC, neural crest, MP, multipotent progenitor, EC, endocardial/endothelial, UN, unknown/unidentified, BL, blood, EP, epicardial, FM, fibromesenchymal, UMI, unique molecular identifiers.

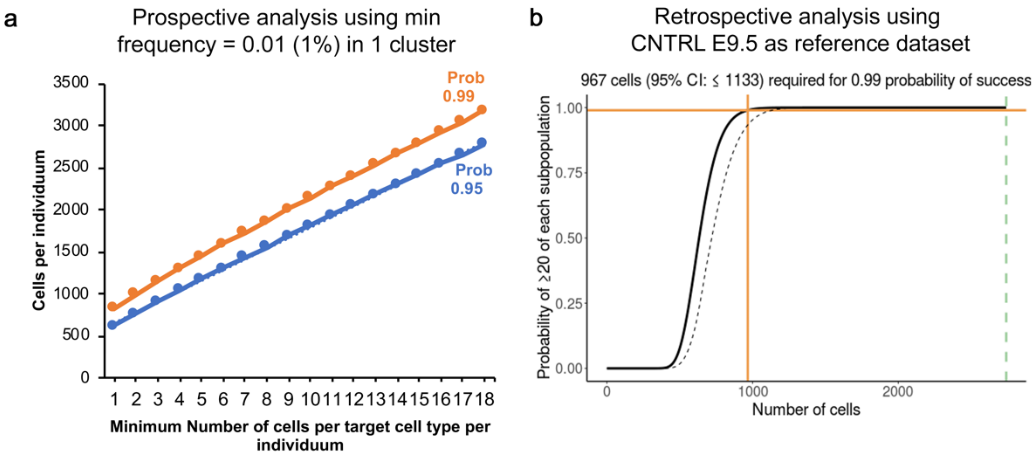

c

Sample	Prospective power analysis	10x Filtered output	Seurat QC filtration	Number of cells in the smallest cell type	Frequency of the smallest cell type	Retrospective power analysis using control
Control E9.5	Expect to detect 15-16 cells for frequency 0.01 when sequencing 2500-3000 cells	3007	2741	90 (immune cells)	0.03	Probability of detecting >= 20 cells of each subpopulation when at least 967(95% CI: <= 1133) cells are sequenced = 0.99
Control E11.5		4350	2780	43 (Ectoderm_endoderm)	0.015	
matHG E9.5		3922	3829	6 (Immune cells)	0.015	
matHG E11.5		3504	2637	24 (Ectoderm_endoderm)	0.01	

Supplementary Figure 4: Power analysis on Single-cell sample size

(a) Statistical power analysis performed using SCOPIT using both prospective and retrospective mode. Graph shows total number of cells in each individual sample (Cells per individuum) required to the minimum number of target cells in a rare cell type (population frequency 0.01 or 1%) in 1 cluster with either 0.99 or 0.95 probability of capture. **(b)** Graph shows the probability of capturing greater than or equal to 20 cells of a rare population with probability of 0.99 with dotted lines showing 95% confidence interval. **(c)** Table listing the results of prospective and retrospective power analysis, and the number of cells identified using the Cell Ranger non-droplets, and filtered cell through tertiary process steps.

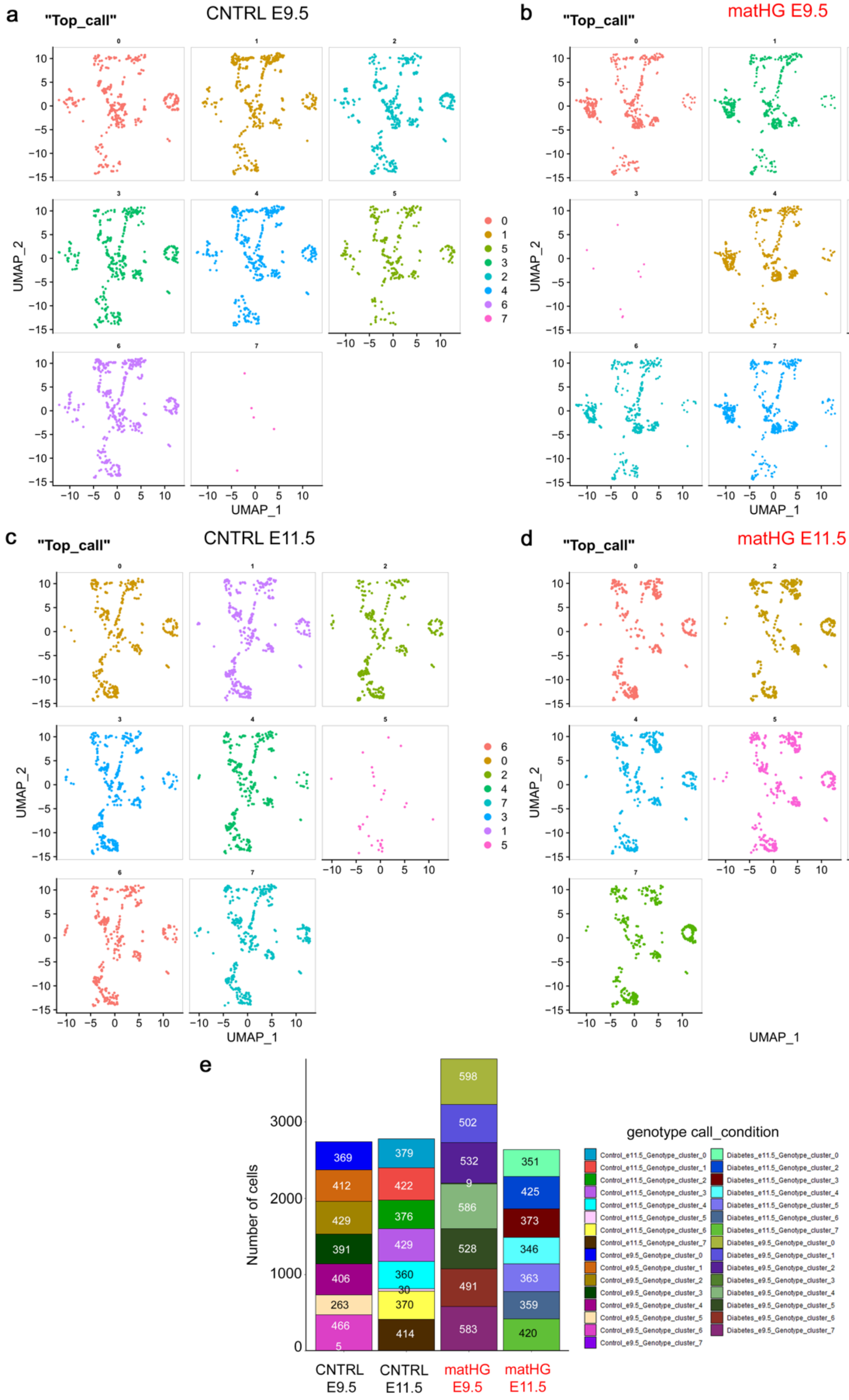
a
"Top_call"
CNTRL E9.5
b
"Top_call"
matHG E9.5
c
"Top_call"
CNTRL E11.5
d
"Top_call"
matHG E11.5
UMAP_1
UMAP_2
e
Number of cells
CNTRL E9.5
CNTRL E11.5
matHG E9.5
matHG E11.5
genotype call_condition
Control_e11.5_Genotype_cluster_0
Control_e11.5_Genotype_cluster_1
Control_e11.5_Genotype_cluster_2
Control_e11.5_Genotype_cluster_3
Control_e11.5_Genotype_cluster_4
Control_e11.5_Genotype_cluster_5
Control_e11.5_Genotype_cluster_6
Control_e11.5_Genotype_cluster_7
Control_e9.5_Genotype_cluster_0
Control_e9.5_Genotype_cluster_1
Control_e9.5_Genotype_cluster_2
Control_e9.5_Genotype_cluster_3
Control_e9.5_Genotype_cluster_4
Control_e9.5_Genotype_cluster_5
Control_e9.5_Genotype_cluster_6
Control_e9.5_Genotype_cluster_7
Diabetes_e11.5_Genotype_cluster_0
Diabetes_e11.5_Genotype_cluster_2
Diabetes_e11.5_Genotype_cluster_3
Diabetes_e11.5_Genotype_cluster_4
Diabetes_e11.5_Genotype_cluster_5
Diabetes_e11.5_Genotype_cluster_6
Diabetes_e11.5_Genotype_cluster_7
Diabetes_e9.5_Genotype_cluster_0
Diabetes_e9.5_Genotype_cluster_1
Diabetes_e9.5_Genotype_cluster_2
Diabetes_e9.5_Genotype_cluster_3
Diabetes_e9.5_Genotype_cluster_4
Diabetes_e9.5_Genotype_cluster_5
Diabetes_e9.5_Genotype_cluster_6
Diabetes_e9.5_Genotype_cluster_7

Supplementary Figure 5: Post-hoc sequencing genotyping of single-cell data

(**a-d**) UMAP plots shown for each sample, generated by adding the predicted genotype calls (0-7) as meta data for each cell in the Seurat object of individual samples. The UMAP plots were split according to the genotype calls (indicated by colors) using split on meta button in Ryabhatta. (**e**) Stacked-bar plot showing the number of cells called for each genotype in CNTRL and matHG-exposed E9.5 and E11.5 hearts using Souporcell. Colors in the bar indicate genotype calls per timepoint per maternal condition. CNTRL, control, matHG, maternal hyperglycemia, UMAP, Uniform manifold approximation and projection.

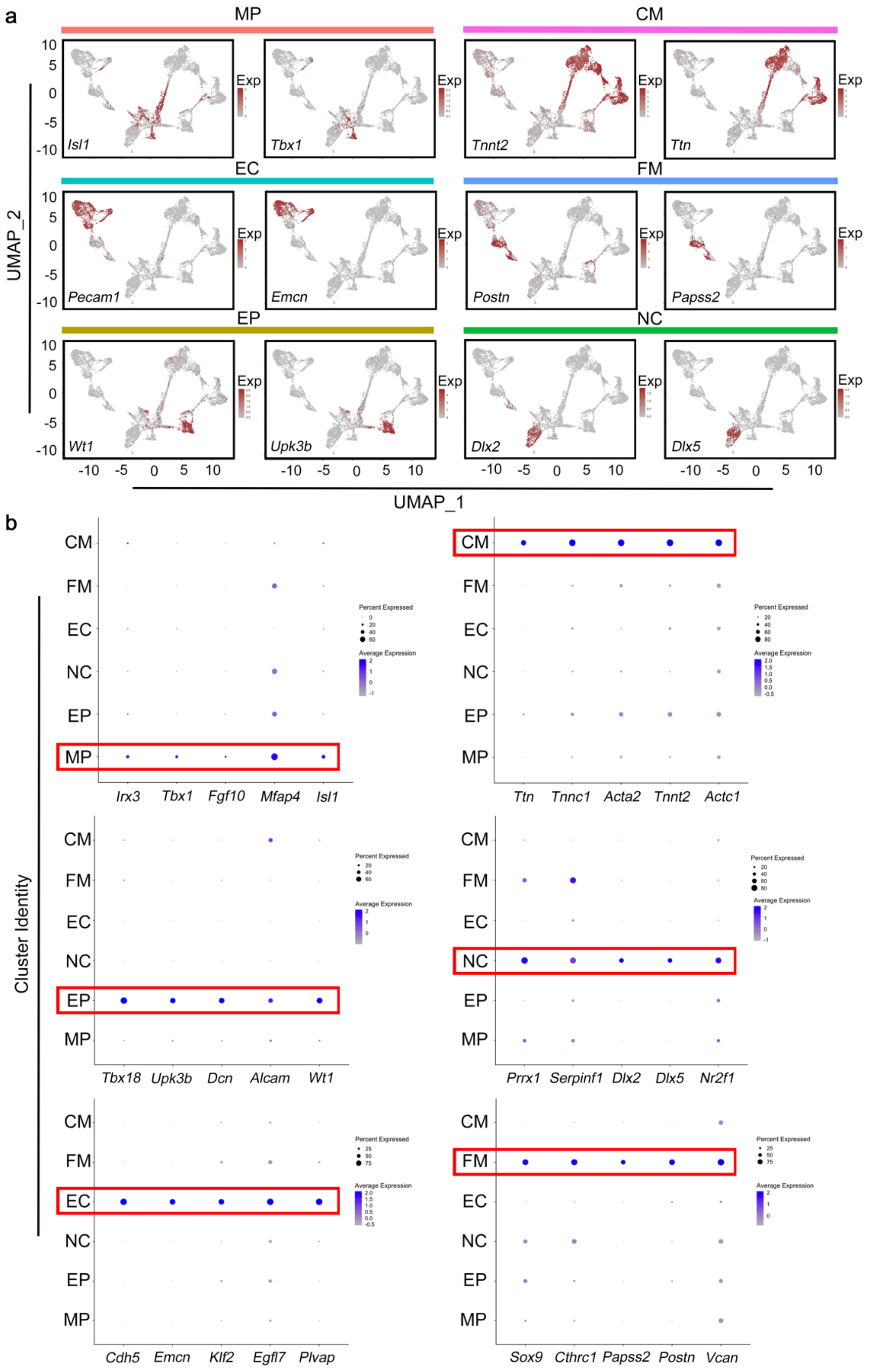
a
MP
CM
EC
FM
EP
NC
Isl1
Tbx1
Tnnt2
Ttn
Pecam1
Emcn
Postn
Papss2
Wt1
Upk3b
Dlx2
Dlx5
Exp
UMAP_2
UMAP_1
b
Cluster Identity
CM
FM
EC
NC
EP
MP
Irx3 Tbx1 Fgf10 Mfap4 Isl1
Ttn Tnnc1 Acta2 Tnnt2 Actc1
Tbx18 Upk3b Dcn Alcam Wt1
Prrx1 Serpinf1 Dlx2 Dlx5 Nr2f1
Cdh5 Emcn Klf2 Egfl7 Plvap
Sox9 Cthrc1 Papss2 Postn Vcan
Percent Expressed
Average Expression

Supplementary Figure 6. Cluster identification based on discrete gene expression profile

(a) UMAP plots illustrate cluster-specific expression of highly expressed marker genes applied to classify six broadly defined cardiac cell populations. The scale indicates Z-scored expression values (red = high expression, grey = no expression). Statistical tests for differential gene-expression applied to 8503 cells. **(b)** Dot plots represent the expression of lineage specific marker genes across MP, EP, NC, EC, FM, and CM clusters. Each dot is sized to represent the percentage of cells of each type expressing the marker gene and colored to represent the average expression of each marker gene across all cells, as shown in the key (dark blue = high expression and light blue = low expression). MP, multipotent progenitor, EP, epicardial, NC, neural crest EC, endocardial/endothelial, FM, fibromesenchymal and CM, cardiomyocytes, UMAP, Uniform manifold approximation and projection.

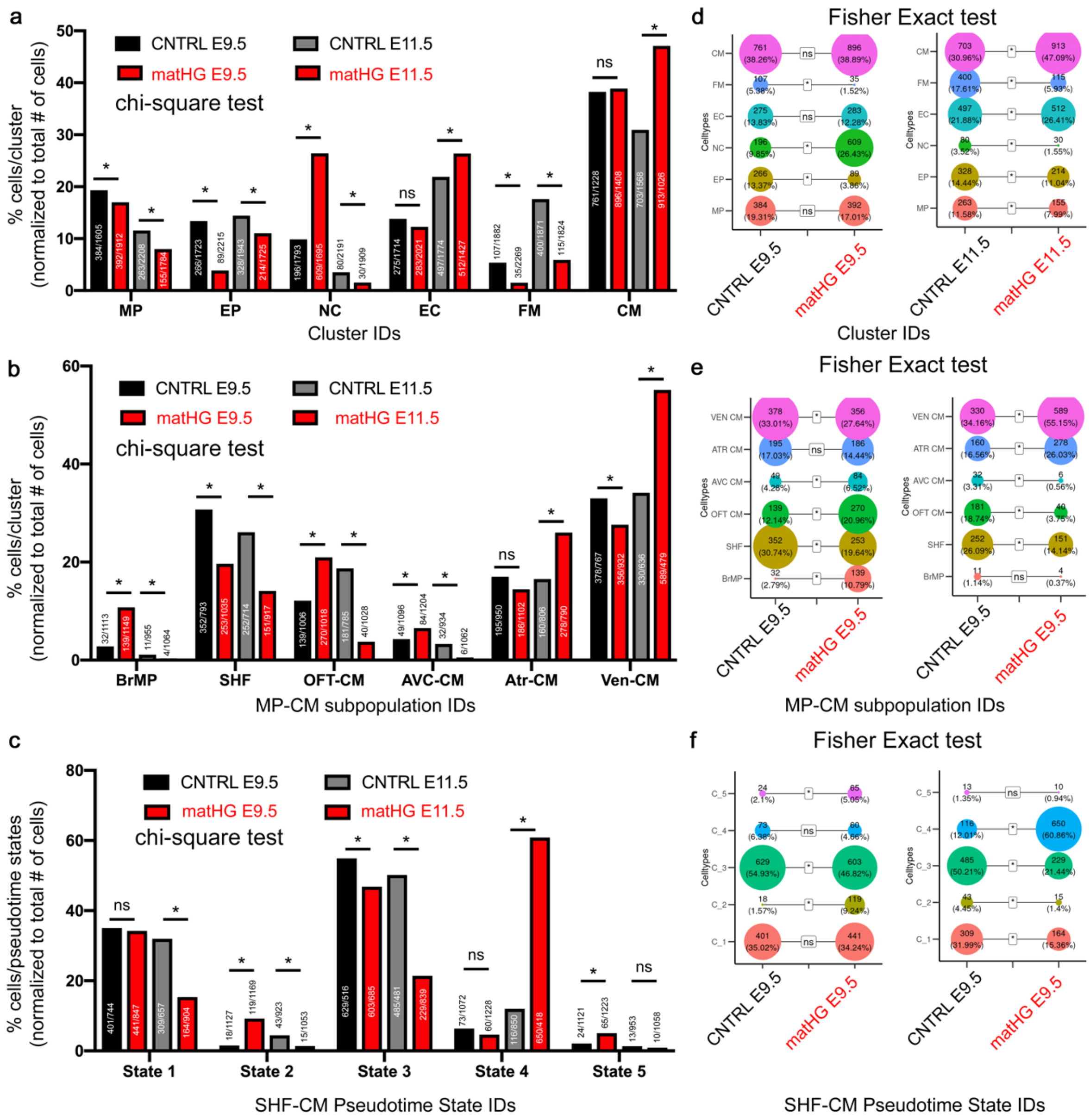

Supplementary Figure 7. Maternal hyperglycemia alters the proportion of cells in each cluster and pseudotime states in developing embryonic hearts

(a-c) Bar graphs show the distribution of cells in six clusters **(a)**, in six MP-CM subclusters **(b)** and in five pseudotime states **(c)** from CNTRL and matHG exposed E9.5 and E11.5 hearts. For cell number comparisons, cells were normalized to the total number of cells analyzed, embedded in the individual bar graphs. Statistical significance between groups

determined by Chi-square test. **(d-f)** Bubble plots show the distribution of cells in six clusters **(d)**, in six MP-CM subclusters **(e)** and in five pseudotime states **(f)** from CNTRL and matHG exposed E9.5 and E11.5 hearts. Statistical significance between groups determined by Fisher Exact test. ns= non-significant and * indicates two-tailed p-value $\leq$ 0.05. CNTRL, control, matHG, maternal hyperglycemia, MP, multipotent progenitor, CM, cardiomyocytes, EP, epicardial, EC, endocardial/endothelial, FM, fibromesenchymal, NC, neural crest, BrMP, branchiomeric muscle progenitors, SHF, second heart field, OFT, outflow tract, AVC, atrioventricular canal, Atr, atrial, Ven, ventricular.

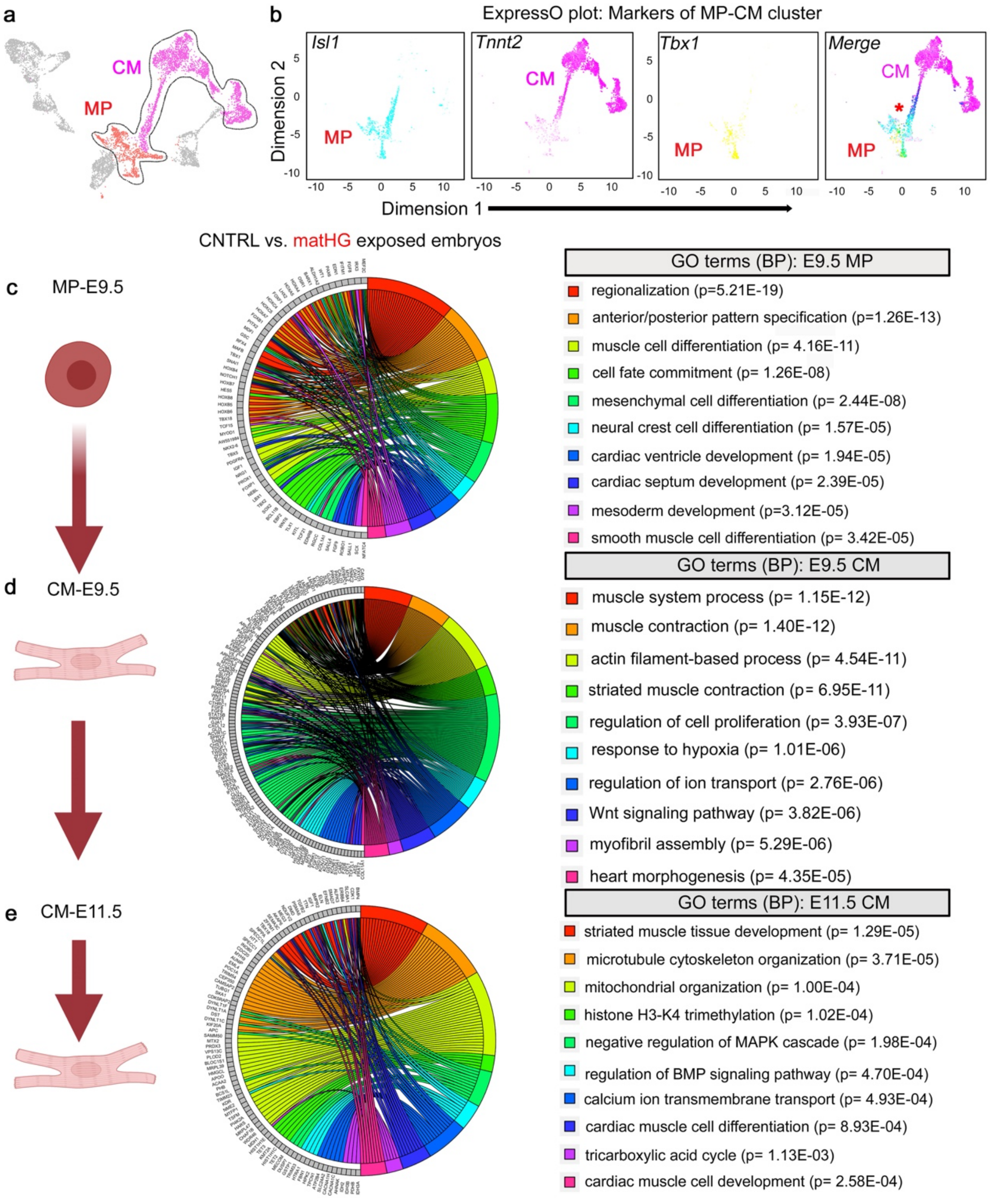
a
CM
MP
b
ExpressO plot: Markers of MP-CM cluster
Isl1
Tnnt2
Tbx1
Merge
Dimension 2
Dimension 1
CM
MP
CNTRL vs. matHG exposed embryos
c
MP-E9.5
d
CM-E9.5
e
CM-E11.5
GO terms (BP): E9.5 MP
regionalization (p=5.21E-19)
anterior/posterior pattern specification (p=1.26E-13)
muscle cell differentiation (p= 4.16E-11)
cell fate commitment (p= 1.26E-08)
mesenchymal cell differentiation (p= 2.44E-08)
neural crest cell differentiation (p= 1.57E-05)
cardiac ventricle development (p= 1.94E-05)
cardiac septum development (p= 2.39E-05)
mesoderm development (p=3.12E-05)
smooth muscle cell differentiation (p= 3.42E-05)
GO terms (BP): E9.5 CM
muscle system process (p= 1.15E-12)
muscle contraction (p= 1.40E-12)
actin filament-based process (p= 4.54E-11)
striated muscle contraction (p= 6.95E-11)
regulation of cell proliferation (p= 3.93E-07)
response to hypoxia (p= 1.01E-06)
regulation of ion transport (p= 2.76E-06)
Wnt signaling pathway (p= 3.82E-06)
myofibril assembly (p= 5.29E-06)
heart morphogenesis (p= 4.35E-05)
GO terms (BP): E11.5 CM
striated muscle tissue development (p= 1.29E-05)
microtubule cytoskeleton organization (p= 3.71E-05)
mitochondrial organization (p= 1.00E-04)
histone H3-K4 trimethylation (p= 1.02E-04)
negative regulation of MAPK cascade (p= 1.98E-04)
regulation of BMP signaling pathway (p= 4.70E-04)
calcium ion transmembrane transport (p= 4.93E-04)
cardiac muscle cell differentiation (p= 8.93E-04)
tricarboxylic acid cycle (p= 1.13E-03)
cardiac muscle cell development (p= 2.58E-04)

Supplementary Figure 8. Transcriptomic analysis of matHG-exposed hearts reveals gene expression differences in cardiac progenitor population and in cardiomyocytes.

(a) Merged UMAP plot demonstrate MP and CM clusters in CNTRL and matHG-exposed E9.5 and E11.5 hearts. Statistical tests for differential gene-expression applied to 4467 cells. **(b)** ExpressO plot show expression of well-known markers of MP (*Isl1* and *Tbx1*) and CM (*Tnnt2*) clusters. * Indicates presence of $Isl1^{+}Tnnt2^{+}$ cells. **(c-e)** GOplots represent the analysis of the gene ontology (GO) terms enriched among the 262 DEGs in E9.5 MP, 357 DEGs in E9.5 CM and 326 DEGs in E11.5 CM clusters (DEG cutoff: Log2Foldchange $\geq$ +1 or $\leq$ -1 and $p_{adjusted} \leq 0.05$). The left side of the circle displays the gene, and the right side shows the GO term associated biological processes. The assorted colors represent different GO terms, and the color of each GO terms is annotated, p-values represent enrichment of GO term. CNTRL, control, matHG, maternal hyperglycemia, MP, multipotent progenitors, CM, cardiomyocytes, DEG, differentially expressed gene, GO, gene ontology, BP, biological processes.

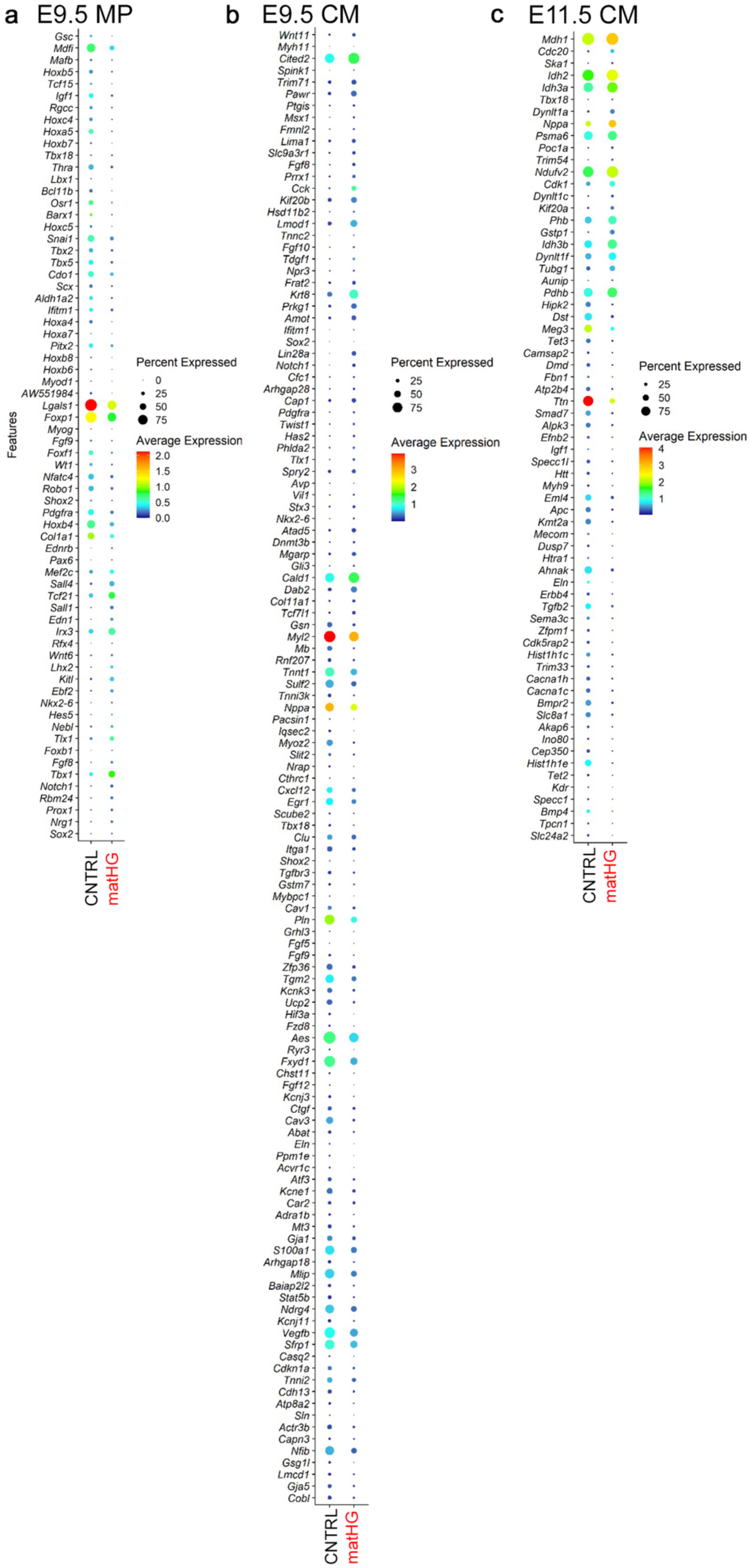
a E9.5 MP
Gsc
Mdfi
Mafb
Hoxb5
Tcf15
Igf1
Rgcc
Hoxc4
Hoxa5
Hoxb7
Tbx18
Thra
Lbx1
Bcl11b
Osr1
Barx1
Hoxc5
Snai1
Tbx2
Tbx5
Cdo1
Scx
Aldh1a2
Ifitm1
Hoxa4
Hoxa7
Pitx2
Hoxb8
Hoxb6
Myod1
AW551984
Lgals1
Foxp1
Myog
Fgf9
Foxf1
Wt1
Nfatc4
Robo1
Shox2
Pdgfra
Hoxb4
Col1a1
Ednrb
Pax6
Mef2c
Sall4
Tcf21
Sall1
Edn1
Irx3
Rfx4
Wnt6
Lhx2
Kitl
Ebf2
Nkx2-6
Hes5
Nebl
Tlx1
Foxb1
Fgf8
Tbx1
Notch1
Rbm24
Prox1
Nrg1
Sox2
Features
CNTRL
matHG
Percent Expressed
0
25
50
75
Average Expression
2.0
1.5
1.0
0.5
0.0
b E9.5 CM
Wnt11
Myh11
Cited2
Spink1
Trim71
Pawr
Ptgis
Msx1
Fmnl2
Lima1
Slc9a3r1
Fgf8
Prrx1
Cck
Kif20b
Hsd11b2
Lmod1
Tnnc2
Fgf10
Tdgf1
Npr3
Frat2
Krt8
Prkg1
Amot
Ifitm1
Sox2
Lin28a
Notch1
Cfc1
Arhgap28
Cap1
Pdgfra
Twist1
Has2
Phlda2
Tlx1
Spry2
Avp
Vil1
Stx3
Nkx2-6
Atad5
Dnmt3b
Mgarp
Gli3
Cald1
Dab2
Col11a1
Tcf7l1
Gsn
Myl2
Mb
Rnf207
Tnnt1
Sulf2
Tnni3k
Nppa
Pacsin1
Iqsec2
Myoz2
Slit2
Nrap
Cthrc1
Cxcl12
Egr1
Scube2
Tbx18
Clu
Itga1
Shox2
Tgfbr3
Gstm7
Mybpc1
Cav1
Pln
Grhl3
Fgf5
Fgf9
Zfp36
Tgm2
Kcnk3
Ucp2
Hif3a
Fzd8
Aes
Ryr3
Fxyd1
Chst11
Fgf12
Kcnj3
Ctgf
Cav3
Abat
Eln
Ppm1e
Acvr1c
Atf3
Kcne1
Car2
Adra1b
Mt3
Gja1
S100a1
Arhgap18
Mlip
Baiap2l2
Stat5b
Ndrg4
Kcnj11
Vegfb
Sfrp1
Casq2
Cdkn1a
Tnni2
Cdh13
Atp8a2
Sln
Actr3b
Capn3
Nfib
Gsg1l
Lmcd1
Gja5
Cobl
CNTRL
matHG
Percent Expressed
25
50
75
Average Expression
3
2
1
c E11.5 CM
Mdh1
Cdc20
Ska1
Idh2
Idh3a
Tbx18
Dynlt1a
Nppa
Psma6
Poc1a
Trim54
Ndufv2
Cdk1
Dynlt1c
Kif20a
Phb
Gstp1
Idh3b
Dynlt1f
Tubg1
Aunip
Pdhb
Hipk2
Dst
Meg3
Tet3
Camsap2
Dmd
Fbn1
Atp2b4
Ttn
Smad7
Alpk3
Efnb2
Igf1
Specc1l
Htt
Myh9
Eml4
Apc
Kmt2a
Mecom
Dusp7
Htra1
Ahnak
Eln
Erbb4
Tgfb2
Sema3c
Zfpm1
Cdk5rap2
Hist1h1c
Trim33
Cacna1h
Cacna1c
Bmpr2
Slc8a1
Akap6
Ino80
Cep350
Hist1h1e
Tet2
Kdr
Specc1
Bmp4
Tpcn1
Slc24a2
CNTRL
matHG
Percent Expressed
25
50
75
Average Expression
4
3
2
1

Supplementary Figure 9. Differentially expressed gene analysis in cardiac progenitor population and in cardiomyocytes.

(a-c) Bubble plots show differentially expressed genes in E9.5 MP, E9.5 and E11.5 CM using DESeq2. The statistical test applied for DESeq2 (pseudobulk) is the Wald test, with the P-value adjusted for multiple testing using the Benjamini and Hochberg method by default. Y-axis label represents genes, and X-axis label maternal diabetic status. Size of the bubble represent percentage of differentially expressed genes enriched in CNTRL vs. matHG-exposed MP and CM. The color of the bubble indicates expression level, where blue and red represents low and high gene expression levels, respectively. Statistical tests for differential gene-expression applied to 4467 cells. CNTRL, control, HG, hyperglycemia, MP, multipotent progenitors, CM, cardiomyocytes.

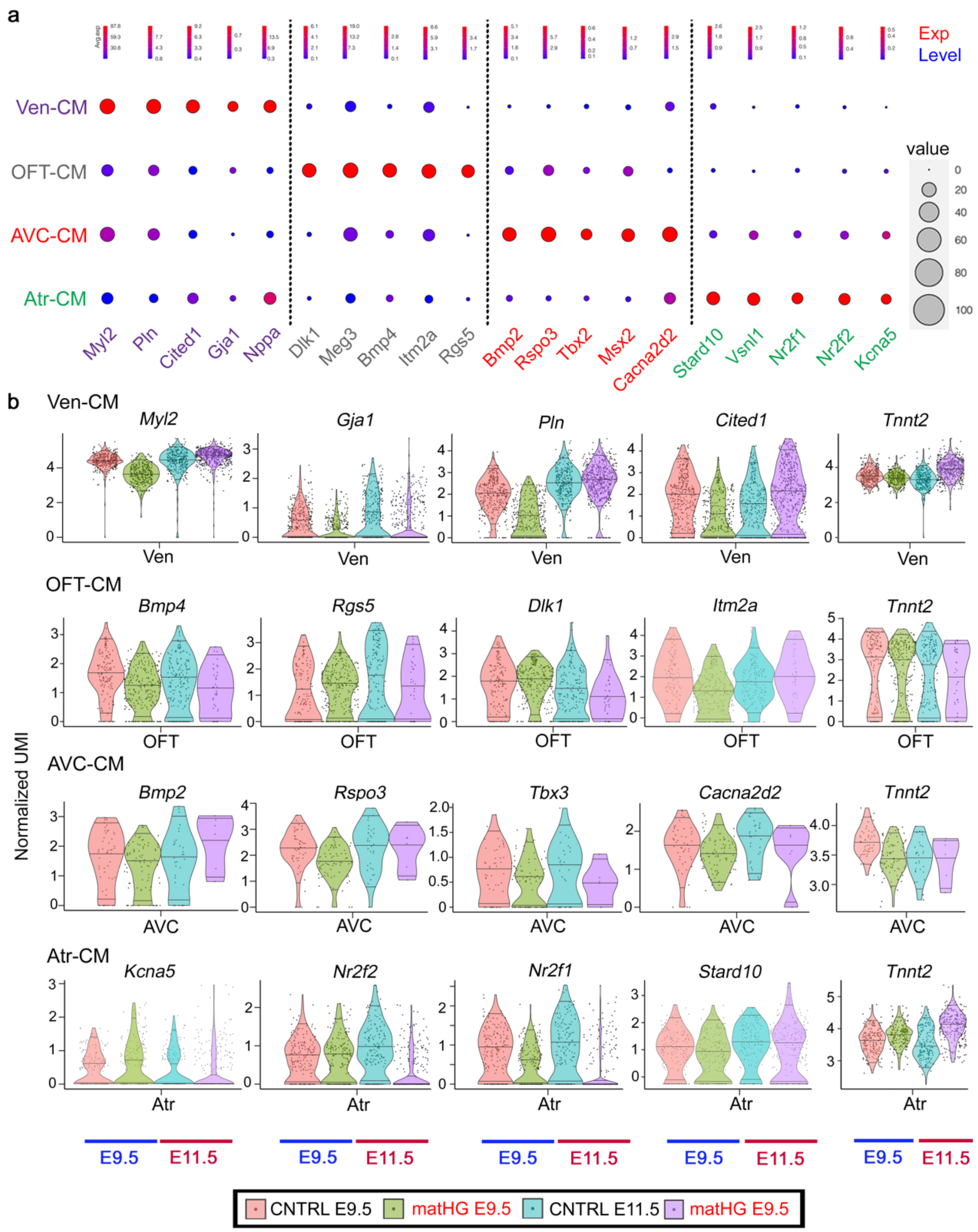
a
Exp
Level
Ven-CM
OFT-CM
AVC-CM
Atr-CM
value
Myl2
Pln
Cited1
Gja1
Nppa
Dlk1
Meg3
Bmp4
Itm2a
Rgs5
Bmp2
Rspo3
Tbx2
Msx2
Cacna2d2
Stard10
Vsnl1
Nr2f1
Nr2f2
Kcna5
b
Ven-CM
Myl2
Gja1
Pln
Cited1
Tnnt2
Ven
OFT-CM
Bmp4
Rgs5
Dlk1
Itm2a
Tnnt2
OFT
AVC-CM
Bmp2
Rspo3
Tbx3
Cacna2d2
Tnnt2
AVC
Atr-CM
Kcna5
Nr2f2
Nr2f1
Stard10
Tnnt2
Atr
Normalized UMI
E9.5
E11.5
CNTRL E9.5
matHG E9.5
CNTRL E11.5
matHG E9.5

Supplementary Figure 10. Impact of maternal hyperglycemia on cardiomyocyte subtypes.

(a) Dot plots represent the expression of known marker genes across CM subtypes (Ven, OFT, AVC, and Atr-CM). Statistical tests for differential gene-expression applied to 3273 cells. Each dot is sized to represent the proportion of cells of each type expressing the marker gene and colored to represent the average expression of each marker gene across all as shown in the key (red = high expression and dark blue = low expression). **(b)** Violin plots demonstrate normalized UMI or gene-expression levels across CM subtypes in CNTRL and matHG-exposed E9.5 and E11.5 hearts. Each dot in the violin plot represents individual cells. CNTRL, control, matHG, maternal hyperglycemia, UMI, unique molecular identifier, Ven, ventricular, OFT, outflow tract, AVC, atrioventricular canal, and Atr, atrial, CM, cardiomyocytes.

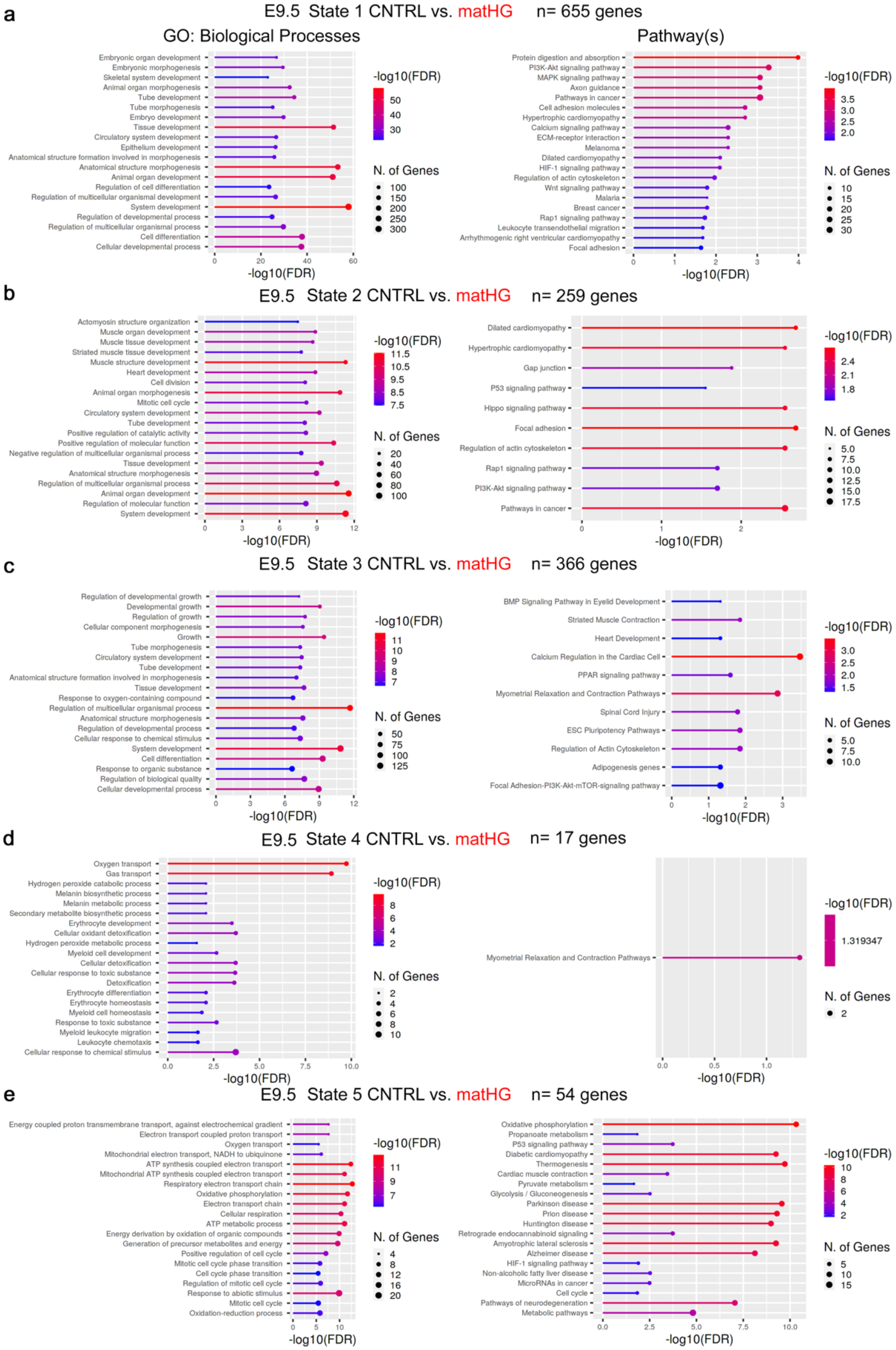
a
E9.5 State 1 CNTRL vs. matHG n= 655 genes
GO: Biological Processes
Pathway(s)
Embryonic organ development
Embryonic morphogenesis
Skeletal system development
Animal organ morphogenesis
Tube development
Tube morphogenesis
Embryo development
Tissue development
Circulatory system development
Epithelium development
Anatomical structure formation involved in morphogenesis
Anatomical structure morphogenesis
Animal organ development
Regulation of cell differentiation
Regulation of multicellular organismal development
System development
Regulation of developmental process
Regulation of multicellular organismal process
Cell differentiation
Cellular developmental process
-log10(FDR)
N. of Genes
Protein digestion and absorption
PI3K-Akt signaling pathway
MAPK signaling pathway
Axon guidance
Pathways in cancer
Cell adhesion molecules
Hypertrophic cardiomyopathy
Calcium signaling pathway
ECM-receptor interaction
Melanoma
Dilated cardiomyopathy
HIF-1 signaling pathway
Regulation of actin cytoskeleton
Wnt signaling pathway
Malaria
Breast cancer
Rap1 signaling pathway
Leukocyte transendothelial migration
Arrhythmogenic right ventricular cardiomyopathy
Focal adhesion
b
E9.5 State 2 CNTRL vs. matHG n= 259 genes
Actomyosin structure organization
Muscle organ development
Muscle tissue development
Striated muscle tissue development
Muscle structure development
Heart development
Cell division
Animal organ morphogenesis
Mitotic cell cycle
Circulatory system development
Tube development
Positive regulation of catalytic activity
Positive regulation of molecular function
Negative regulation of multicellular organismal process
Tissue development
Anatomical structure morphogenesis
Regulation of multicellular organismal process
Animal organ development
Regulation of molecular function
System development
Dilated cardiomyopathy
Hypertrophic cardiomyopathy
Gap junction
P53 signaling pathway
Hippo signaling pathway
Focal adhesion
Regulation of actin cytoskeleton
Rap1 signaling pathway
PI3K-Akt signaling pathway
Pathways in cancer
c
E9.5 State 3 CNTRL vs. matHG n= 366 genes
Regulation of developmental growth
Developmental growth
Regulation of growth
Cellular component morphogenesis
Growth
Tube morphogenesis
Circulatory system development
Tube development
Anatomical structure formation involved in morphogenesis
Tissue development
Response to oxygen-containing compound
Regulation of multicellular organismal process
Anatomical structure morphogenesis
Regulation of developmental process
Cellular response to chemical stimulus
System development
Cell differentiation
Response to organic substance
Regulation of biological quality
Cellular developmental process
BMP Signaling Pathway in Eyelid Development
Striated Muscle Contraction
Heart Development
Calcium Regulation in the Cardiac Cell
PPAR signaling pathway
Myometrial Relaxation and Contraction Pathways
Spinal Cord Injury
ESC Pluripotency Pathways
Regulation of Actin Cytoskeleton
Adipogenesis genes
Focal Adhesion-PI3K-Akt-mTOR-signaling pathway
d
E9.5 State 4 CNTRL vs. matHG n= 17 genes
Oxygen transport
Gas transport
Hydrogen peroxide catabolic process
Melanin biosynthetic process
Melanin metabolic process
Secondary metabolite biosynthetic process
Erythrocyte development
Cellular oxidant detoxification
Hydrogen peroxide metabolic process
Myeloid cell development
Cellular detoxification
Cellular response to toxic substance
Detoxification
Erythrocyte differentiation
Erythrocyte homeostasis
Myeloid cell homeostasis
Response to toxic substance
Myeloid leukocyte migration
Leukocyte chemotaxis
Cellular response to chemical stimulus
Myometrial Relaxation and Contraction Pathways
1.319347
e
E9.5 State 5 CNTRL vs. matHG n= 54 genes
Energy coupled proton transmembrane transport, against electrochemical gradient
Electron transport coupled proton transport
Oxygen transport
Mitochondrial electron transport, NADH to ubiquinone
ATP synthesis coupled electron transport
Mitochondrial ATP synthesis coupled electron transport
Respiratory electron transport chain
Oxidative phosphorylation
Electron transport chain
Cellular respiration
ATP metabolic process
Energy derivation by oxidation of organic compounds
Generation of precursor metabolites and energy
Positive regulation of cell cycle
Mitotic cell cycle phase transition
Cell cycle phase transition
Regulation of mitotic cell cycle
Response to abiotic stimulus
Mitotic cell cycle
Oxidation-reduction process
Oxidative phosphorylation
Propanoate metabolism
P53 signaling pathway
Diabetic cardiomyopathy
Thermogenesis
Cardiac muscle contraction
Pyruvate metabolism
Glycolysis / Gluconeogenesis
Parkinson disease
Prion disease
Huntington disease
Retrograde endocannabinoid signaling
Amyotrophic lateral sclerosis
Alzheimer disease
HIF-1 signaling pathway
Non-alcoholic fatty liver disease
MicroRNAs in cancer
Cell cycle
Pathways of neurodegeneration
Metabolic pathways

Supplementary Figure 11. Transcriptional changes in MP-CM subpopulations at E9.5 across the pseudotime trajectories

(a-e) Lollipop charts illustrate the top 20 GO-terms for Biological Processes and Pathways, sorted as descending negative logarithmic adjusted p-value of enrichment analysis (-log10(FDR)) shown in the key. Numbers in circles represent the number of DEGs matched to a specific GO term. Five pseudotime states were compared between CNTRL and matHG exposed E9.5 MP-CM subclusters. GO, gene ontology, FDR, false discovery rate, DEGs, differentially expressed genes, CNTRL, control, matHG, maternal hyperglycemia.

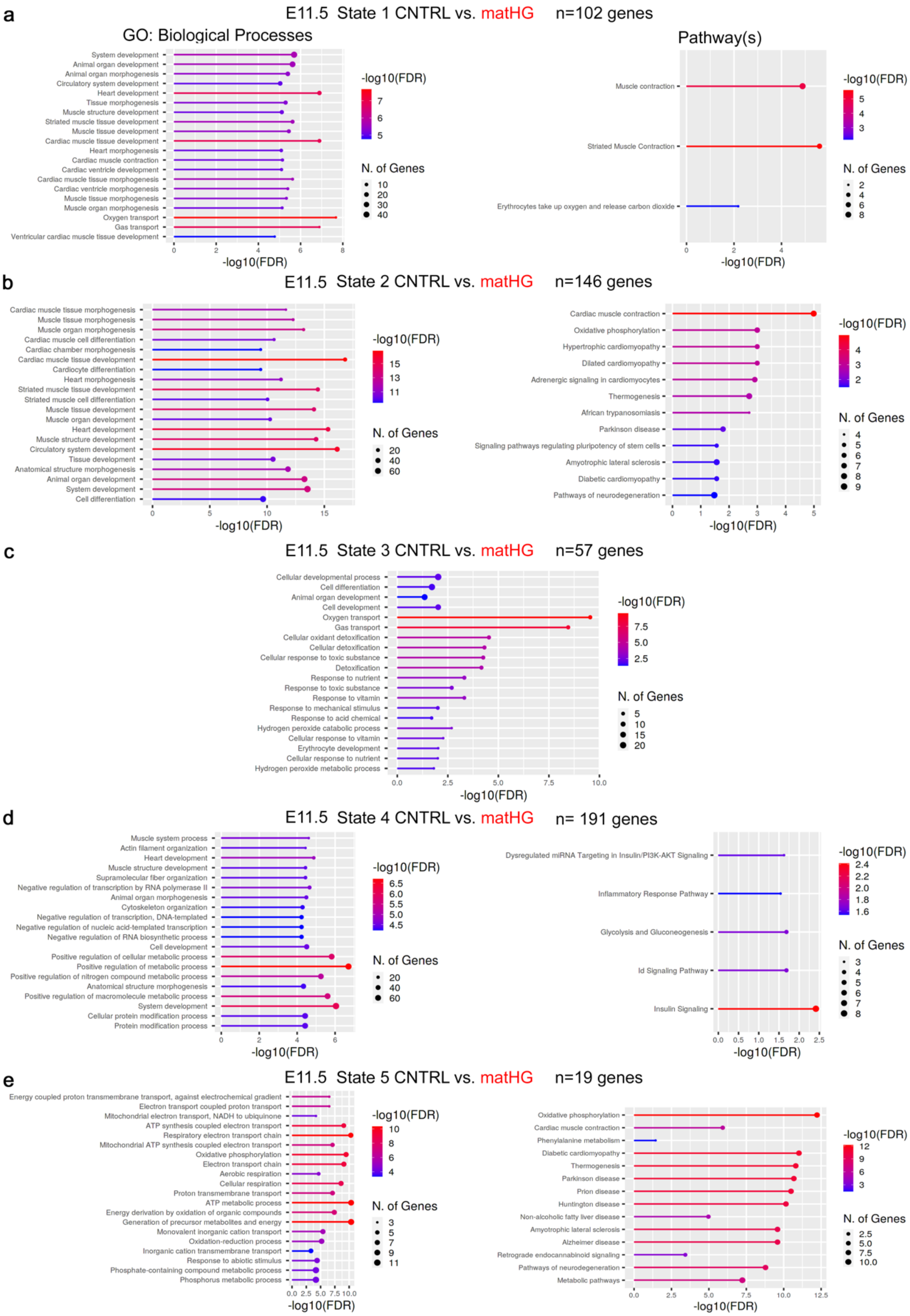
a
E11.5 State 1 CNTRL vs. matHG n=102 genes
GO: Biological Processes
System development
Animal organ development
Animal organ morphogenesis
Circulatory system development
Heart development
Tissue morphogenesis
Muscle structure development
Striated muscle tissue development
Muscle tissue development
Cardiac muscle tissue development
Heart morphogenesis
Cardiac muscle contraction
Cardiac ventricle development
Cardiac muscle tissue morphogenesis
Cardiac ventricle morphogenesis
Muscle tissue morphogenesis
Muscle organ morphogenesis
Oxygen transport
Gas transport
Ventricular cardiac muscle tissue development
-log10(FDR)
N. of Genes
Pathway(s)
Muscle contraction
Striated Muscle Contraction
Erythrocytes take up oxygen and release carbon dioxide
-log10(FDR)
N. of Genes
b
E11.5 State 2 CNTRL vs. matHG n=146 genes
Cardiac muscle tissue morphogenesis
Muscle tissue morphogenesis
Muscle organ morphogenesis
Cardiac muscle cell differentiation
Cardiac chamber morphogenesis
Cardiac muscle tissue development
Cardiocyte differentiation
Heart morphogenesis
Striated muscle tissue development
Striated muscle cell differentiation
Muscle tissue development
Muscle organ development
Heart development
Muscle structure development
Circulatory system development
Tissue development
Anatomical structure morphogenesis
Animal organ development
System development
Cell differentiation
-log10(FDR)
N. of Genes
Cardiac muscle contraction
Oxidative phosphorylation
Hypertrophic cardiomyopathy
Dilated cardiomyopathy
Adrenergic signaling in cardiomyocytes
Thermogenesis
African trypanosomiasis
Parkinson disease
Signaling pathways regulating pluripotency of stem cells
Amyotrophic lateral sclerosis
Diabetic cardiomyopathy
Pathways of neurodegeneration
-log10(FDR)
N. of Genes
c
E11.5 State 3 CNTRL vs. matHG n=57 genes
Cellular developmental process
Cell differentiation
Animal organ development
Cell development
Oxygen transport
Gas transport
Cellular oxidant detoxification
Cellular detoxification
Cellular response to toxic substance
Detoxification
Response to nutrient
Response to toxic substance
Response to vitamin
Response to mechanical stimulus
Response to acid chemical
Hydrogen peroxide catabolic process
Cellular response to vitamin
Erythrocyte development
Cellular response to nutrient
Hydrogen peroxide metabolic process
-log10(FDR)
N. of Genes
d
E11.5 State 4 CNTRL vs. matHG n= 191 genes
Muscle system process
Actin filament organization
Heart development
Muscle structure development
Supramolecular fiber organization
Negative regulation of transcription by RNA polymerase II
Animal organ morphogenesis
Cytoskeleton organization
Negative regulation of transcription, DNA-templated
Negative regulation of nucleic acid-templated transcription
Negative regulation of RNA biosynthetic process
Cell development
Positive regulation of cellular metabolic process
Positive regulation of metabolic process
Positive regulation of nitrogen compound metabolic process
Anatomical structure morphogenesis
Positive regulation of macromolecule metabolic process
System development
Cellular protein modification process
Protein modification process
-log10(FDR)
N. of Genes
Dysregulated miRNA Targeting in Insulin/PI3K-AKT Signaling
Inflammatory Response Pathway
Glycolysis and Gluconeogenesis
Id Signaling Pathway
Insulin Signaling
-log10(FDR)
N. of Genes
e
E11.5 State 5 CNTRL vs. matHG n=19 genes
Energy coupled proton transmembrane transport, against electrochemical gradient
Electron transport coupled proton transport
Mitochondrial electron transport, NADH to ubiquinone
ATP synthesis coupled electron transport
Respiratory electron transport chain
Mitochondrial ATP synthesis coupled electron transport
Oxidative phosphorylation
Electron transport chain
Aerobic respiration
Cellular respiration
Proton transmembrane transport
ATP metabolic process
Energy derivation by oxidation of organic compounds
Generation of precursor metabolites and energy
Monovalent inorganic cation transport
Oxidation-reduction process
Inorganic cation transmembrane transport
Response to abiotic stimulus
Phosphate-containing compound metabolic process
Phosphorus metabolic process
-log10(FDR)
N. of Genes
Oxidative phosphorylation
Cardiac muscle contraction
Phenylalanine metabolism
Diabetic cardiomyopathy
Thermogenesis
Parkinson disease
Prion disease
Huntington disease
Non-alcoholic fatty liver disease
Amyotrophic lateral sclerosis
Alzheimer disease
Retrograde endocannabinoid signaling
Pathways of neurodegeneration
Metabolic pathways
-log10(FDR)
N. of Genes

Supplementary Figure 12. Transcriptomic changes in MP-CM subpopulations at E11.5 across the pseudotime trajectories

(a-e) Lollipop plots represent the top 20 GO-terms for Biological Processes and Pathways, sorted as descending negative logarithmic adjusted p-value of enrichment analysis (-log10(FDR)) shown in the key. Numbers in circles represent the number of DEGs matched to a specific GO term. Five pseudotime states were compared between CNTRL and matHG exposed E11.5 MP-CM subclusters. No significantly enriched pathways were noted in cells present in State 3 at E11.5. GO, gene ontology, FDR, false discovery rate, DEGs, differentially expressed genes, CNTRL, control, matHG, maternal hyperglycemia.

226

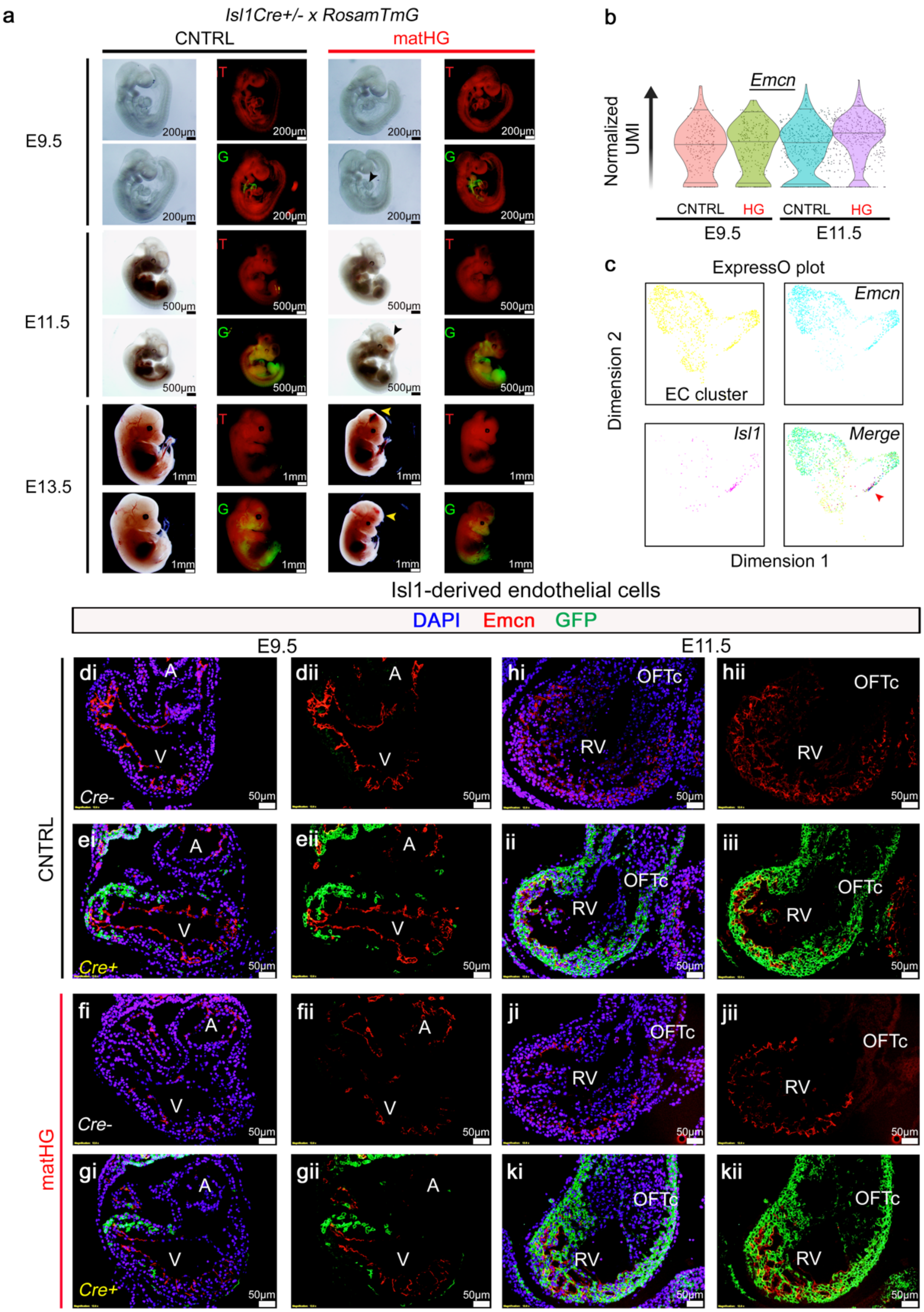
a
Isl1Cre+/- x RosamTmG
CNTRL
matHG
E9.5
E11.5
E13.5
b
Emcn
Normalized UMI
CNTRL HG CNTRL HG
E9.5 E11.5
c
ExpressO plot
EC cluster
Emcn
Isl1
Merge
Dimension 2
Dimension 1
Isl1-derived endothelial cells
DAPI Emcn GFP
E9.5
E11.5
CNTRL
matHG
Cre-
Cre+
A
V
OFTc
RV

Supplementary Figure 13. *Isl1*-cell fate mapping studies under maternal hyperglycemic exposure.

(a) Representative whole-mount brightfield and fluorescence images of *Isl1Cre⁻; Rosa*$^{mTmG/+}$ (T; red, tdTomato^{+}) and *Isl1Cre*$^{+}$*; Rosa*$^{mTmG/+}$ (G; green, GFP^{+}) littermate control hearts at E9.5, E11.5 and E13.5 exposed to CNTRL and matHG environment. GFP^{+} expression demonstrate the endogenous pattern of *Isl1* expression, and those genetically labelled descendants derived from *Isl1* progenitors. MatHG-exposed *Isl1Cre*$^{+}$*; Rosa*$^{mTmG/+}$ embryos show intracerebral hemorrhage and exencephaly at E11.5 and E13.5 (shown in black and yellow arrowheads). **(b)** Violin plots show normalized UMI or expression of *Emcn*, an endothelial cell marker, in E9.5 and E11.5 scRNA-seq data. **(c)** ExpressO plots display *Isl1*$^{+}$*Emcn*$^{+}$ cells in EC cluster (red arrowheads). **(d-k)** Representative immunofluorescent images show GFP (green) and Emcn (red) protein expression in CNTRL and matHG-exposed Cre- and Cre+ littermate controls (n$\geq$2 embryos/timepoint/ maternal condition). Nuclei stained with DAPI shown in blue. *Isl1*-derived Emcn^{+}GFP^{+} ECs shown in yellow. CNTRL, control, matHG, maternal hyperglycemia, EC, endocardial/endothelial. A, atria, V, ventricle, OFTc, outflow tract cushion, RV, right ventricle. Scale bars: 200µm (E9.5), 500µm (E11.5) and 1mm (E13.5) **(a)** and 50µm **(d-k)**.

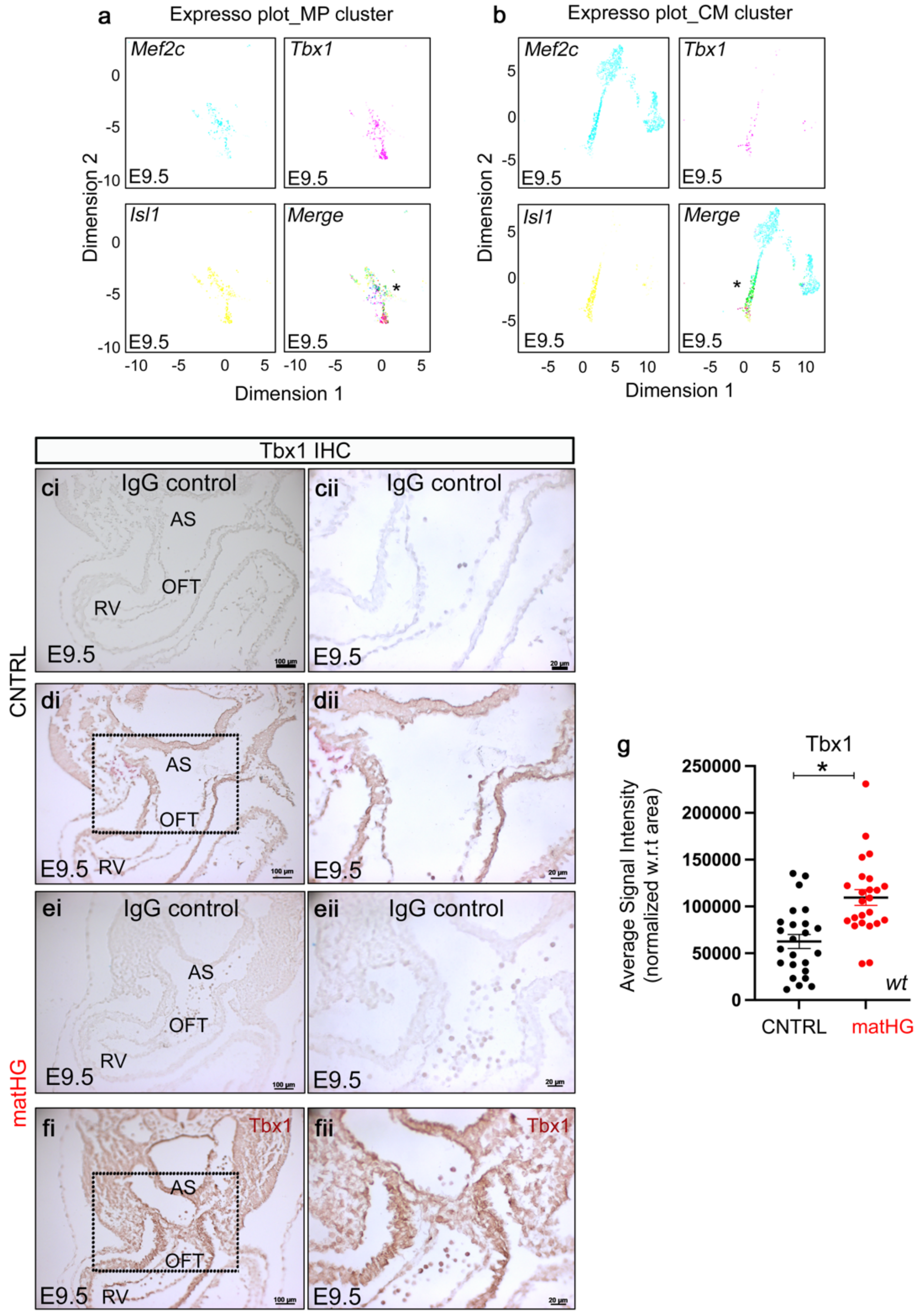
a
Expresso plot_MP cluster
Mef2c
Tbx1
Isl1
Merge
E9.5
Dimension 2
Dimension 1
b
Expresso plot_CM cluster
Tbx1 IHC
ci
IgG control
cii
AS
OFT
RV
CNTRL
di
dii
ei
eii
matHG
fi
fii
Tbx1
g
Average Signal Intensity
(normalized w.r.t area)
250000
200000
150000
100000
50000
0
*
wt
CNTRL
matHG

Supplementary Figure 14. Perturbation in disease associated cardiac progenitor marker, Tbx1 in response to maternal hyperglycemia.

(a, b) ExpressO plots show transcript expression of *Mef2c, Tbx1,* and *Isl1* in MP and CM clusters at E9.5. Black asterisks indicate co-expression of these SHF progenitor markers. **(c-f)** Immunohistochemical staining of transverse cardiac sections with Tbx1 in CNTRL vs. matHG exposed E9.5 *wt* hearts (n=3 independent embryos/maternal diabetes condition). **cii-fii** show higher magnification images of **ci-fi** (indicated by black rectangular boxes). **ci** and **ei** represent rabbit IgG (negative) control. **(g)** Quantification of Tbx1 protein expression in CNTRL and matHG-exposed E9.5 hearts. Statistical comparisons made between CNTRL and matHG groups by unpaired *t*-test with Welch's correction using GraphPad Prism 9. Data presented as mean ± SEM. * Indicates two-tailed p-value < 0.05. MP, multipotent progenitors, CM, cardiomyocytes, CNTRL, control, matHG, maternal hyperglycemia, wt, wildtype, OFT, outflow tract, AS, aortic sac, IHC, immunohistochemistry. Scale bars: 100µm (**ci-fi**) and 20µm (**cii-fii**).

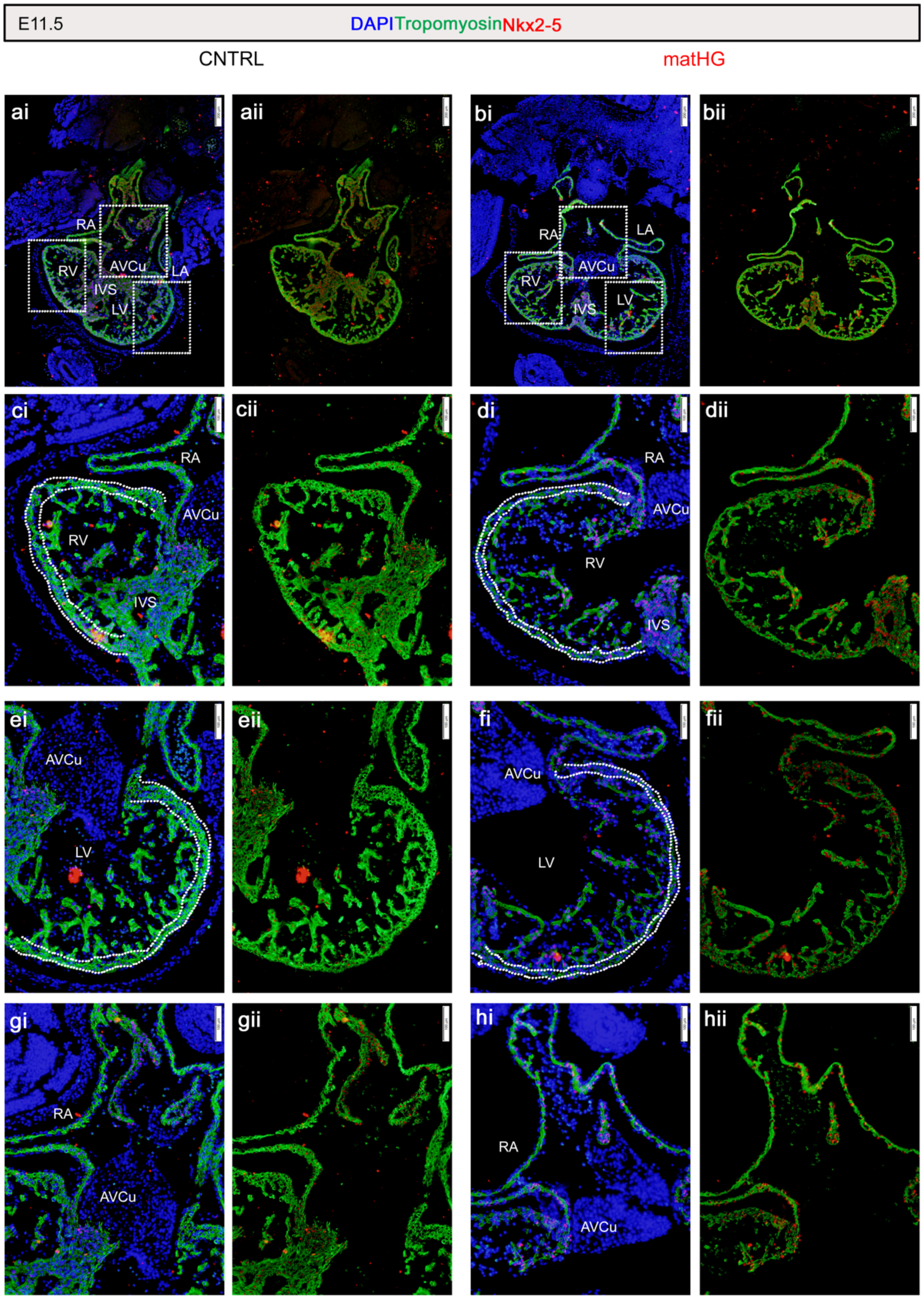
E11.5
DAPITropomyosinNkx2-5
CNTRL
matHG
ai
RA
RV
AVCu
LA
IVS
LV
aii
bi
RA
LA
RV
AVCu
LV
IVS
bii
ci
RA
AVCu
RV
IVS
cii
di
RA
AVCu
RV
IVS
dii
ei
AVCu
LV
eii
fi
AVCu
LV
fii
gi
RA
AVCu
gii
hi
RA
AVCu
hii

Supplementary Figure 15. Maternal hyperglycemic exposure affects the sarcomeric protein expression in E11.5 cardiomyocytes.

(a-h) Representative immunofluorescence images of CNTRL and matHG-exposed E11.5 hearts (n=2 independent embryos per group) labeled with sarcomeric protein, Tropomyosin and CM marker, Nkx2-5. TPM1+Nkx2-5+ expression found in both the atria and ventricles at the timepoint of analysis and demonstrate a decrease upon matHG exposure. C-H, higher magnification images of A and B, indicated by white boxes. Dotted white lines in C-F show thin myocardial wall and impaired trabeculation in matHG-exposed hearts. CM, cardiomyocytes, CNTRL, control, matHG, maternal hyperglycemia, wt, wildtype, RV, right ventricle, LV, left ventricle, RA, right atria, LA, left atria, IVS, interventricular septum, AVCu, atrioventricular cushion. Scale bars: 200μm (**ai, aii, bi, bii**) and 100 μm (**ci-hi, and cii-hii**).

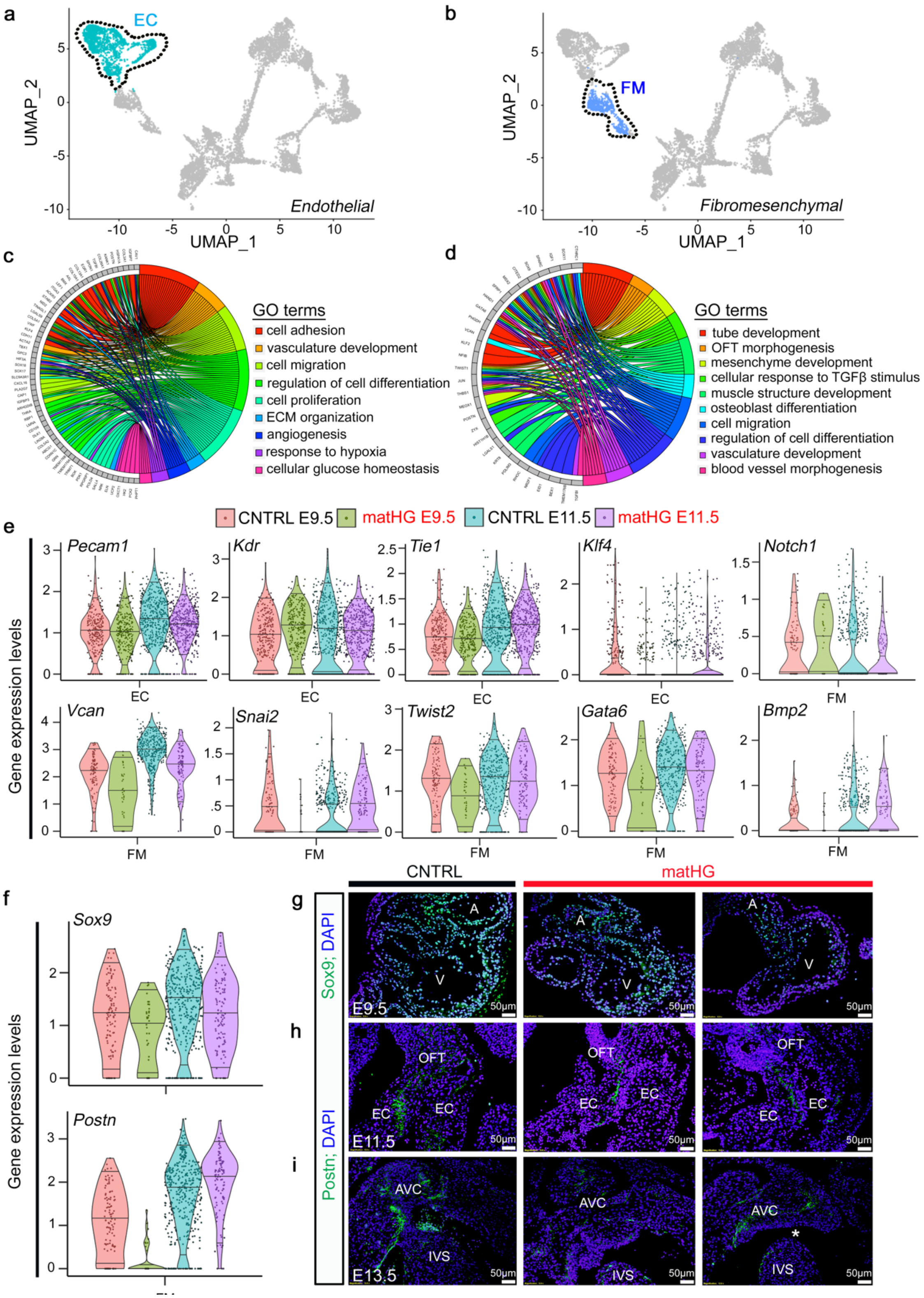
a
EC
UMAP_2
UMAP_1
Endothelial
b
FM
Fibromesenchymal
c
GO terms
cell adhesion
vasculature development
cell migration
regulation of cell differentiation
cell proliferation
ECM organization
angiogenesis
response to hypoxia
cellular glucose homeostasis
d
GO terms
tube development
OFT morphogenesis
mesenchyme development
cellular response to TGFβ stimulus
muscle structure development
osteoblast differentiation
cell migration
regulation of cell differentiation
vasculature development
blood vessel morphogenesis
e
CNTRL E9.5
matHG E9.5
CNTRL E11.5
matHG E11.5
Gene expression levels
Pecam1
Kdr
Tie1
Klf4
Notch1
EC
FM
Vcan
Snai2
Twist2
Gata6
Bmp2
f
Sox9
Postn
Gene expression levels
CNTRL
matHG
g
h
i
Sox9; DAPI
Postn; DAPI
A
V
E9.5
OFT
EC
E11.5
AVC
IVS
E13.5
*
50µm

Supplementary Figure 16. Single-cell RNA-seq reveals transcriptional changes in endocardial/endothelial and mesenchymal cells exposed to maternal hyperglycemia

(a, b) UMAP plots represent the EC and FM clusters from E9.5 and E11.5 embryonic hearts subjected to CNTRL and matHG. **(c, d)** GOplots represent the analysis of the GO terms enriched among the DEGs in E9.5 and E11.5 EC and FM clusters (DEG cutoff: Log2Foldchange $\geq$ 1 or $\leq$ -1 and $P_{adjusted} \leq 0.05$). The left side of the circle displays the gene, and the right side shows the GO-term associated biological processes. The assorted colors represent different GO terms. **(e)** Violin plots show the normalized expression levels of highly variable genes in EC (*Pecam1, Kdr, Tie1, and Klf4*) and FM (*Vcan, Snai2, Twist2, Gata6, Bmp2, and Notch1*) clusters from CNTRL and matHG-exposed E9.5 and E11.5 embryonic hearts. **(f)** Violin plots illustrating *Sox9* and *Postn* gene-expression in E9.5 and E11.5 FM populations exposed to CNTRL and matHG environment. **(g-i)** Panels of immunofluorescent images show protein expression of Sox9 (green) and Postn (green) at E9.5, E11.5 and E13.5 hearts exposed to CNTRL and matHG. Nuclei stained with DAPI shown in blue. White asterisk denotes presence of VSD in matHG-exposed E13.5 embryo. Nuclei stained with DAPI in blue. CNTRL, control, HG, hyperglycemia, GO, gene ontology, FM, fibromesenchymal, A, atria; V, ventricle; OFT, outflow tract; EC, endocardial/endothelial cushion; AVC, atrioventricular canal; IVS, interventricular septum, VSD, ventricular septal defect. Scale bars: **g-i**: 50µm.

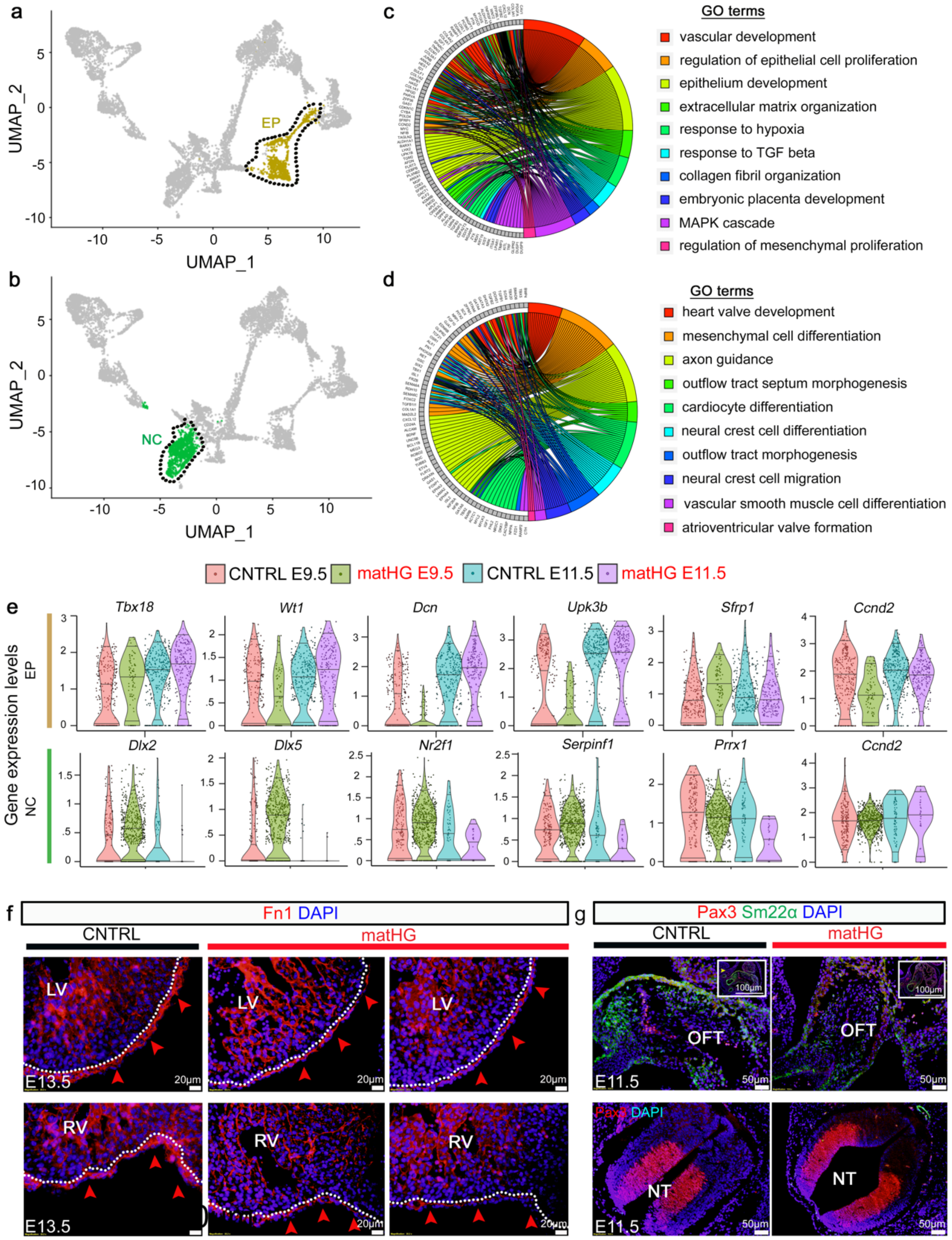
a
EP
UMAP_1
UMAP_2
b
NC
UMAP_1
UMAP_2
c
GO terms
vascular development
regulation of epithelial cell proliferation
epithelium development
extracellular matrix organization
response to hypoxia
response to TGF beta
collagen fibril organization
embryonic placenta development
MAPK cascade
regulation of mesenchymal proliferation
d
GO terms
heart valve development
mesenchymal cell differentiation
axon guidance
outflow tract septum morphogenesis
cardiocyte differentiation
neural crest cell differentiation
outflow tract morphogenesis
neural crest cell migration
vascular smooth muscle cell differentiation
atrioventricular valve formation
CNTRL E9.5
matHG E9.5
CNTRL E11.5
matHG E11.5
e
Gene expression levels
EP
Tbx18
Wt1
Dcn
Upk3b
Sfrp1
Ccnd2
NC
Dlx2
Dlx5
Nr2f1
Serpinf1
Prrx1
Ccnd2
f
Fn1 DAPI
CNTRL
matHG
LV
RV
E13.5
20µm
g
Pax3 Sm22α DAPI
CNTRL
matHG
OFT
100µm
50µm
E11.5
Pax3 DAPI
NT

Supplementary Figure 17. Single-cell RNA-seq reveals transcriptional changes in epicardial and cardiac neural crest cell populations exposed to maternal hyperglycemia

(a, b) UMAP plots represent the EP and NC clusters from E9.5 and E11.5 embryonic hearts subjected to CNTRL and matHG. **(c, d)** GOplots represent the GO terms enriched among the DEGs in E9.5 and E11.5 EP and NC clusters (DEG cutoff: Log2Foldchange $\geq$ 1 or $\leq$ -1 and $P_{adjusted} \leq 0.05$). The left-side of the circle displays the gene, and the right-side shows GO-term associated biological processes. The assorted colors represent GO terms. **(e)** Violin plots show the normalized expression levels of highly variable genes in EP (*Tbx18, Wt1, Dcn, Upk3b, Sfrp1, and Ccnd2*) and NC (*Dlx2, Dlx5, Nr2f1, Serpinf1, Prrx1, and Ccnd2*) clusters from CNTRL and matHG-exposed E9.5 and E11.5 embryonic hearts. **(f)** Panels of immunofluorescent images show protein expression of *Fn1* (red arrowheads) in the EP from low to high-matHG exposed E13.5 LV and RV compared to CNTRL (indicated by dotted white line). **(g)** Panels of immunofluorescent images illustrate the protein expression of NC-markers, Pax3 (red) and Sm22α (green) in E11.5 OFT exposed to matHG vs. CNTRL. Insets (white square boxes) show positive Pax3 expression in the NT. Nuclei stained with DAPI in blue. CNTRL, control, HG, hyperglycemia, GO, gene ontology, EP, epicardial, NC, neural crest, LV, left ventricle, RV, right ventricle, OFT, outflow tract, NT, neural tube. Scale bars: **f**: 20µm and **g**:50µm.

Supplementary Tables:

Supplementary Table 1: Distribution of *Isl1-Cre*$^{+/-}$*; Rosa*$^{mT/mG}$ embryos in the setting of control and matHG environment

Maternal Status	Maternal B.G ± SD (litters)$^{\#}$	Embryonic timepoint	Number of embryos	*Rosa*$^{mT/mG}$*; Cre-*	*Rosa*$^{mT/mG}$*; Cre+*	2-tailed P value (χ^2 test) *
CNTRL	220.7 ± 22.4 (3)	E9.5	28	11	17	0.5908
matHG	335.0 ± 58.7 (3)		28	14	14	
CNTRL	205.3 ± 37.8 (3)	E11.5	22	14	8	0.2949
matHG	420.8 ±191.8 (4)		33	15	18	
CNTRL	244.7 ± 25.0 (3)	E13.5	26	13	13	0.7930
matHG	624.0 ± 66.7 (4)		28	15	13	

$^{\#}$ Unpaired t test indicates statistically significant differences in maternal B.G. at each developmental stage.

*Chi-square test with Yates's correction indicates differences between number of embryos of each genotype recovered from *Isl1-Cre*$^{+/-}$ and homozygous *Rosa*$^{mT/mG}$ crossings.

Supplementary Table 2. Oligonucleotide sequences

Primer Name	Forward Primer	Reverse Primer
GFP	CTGCTGCCCGACAACCAC	TGTGATCGCGCTTCTCGTT
Isl1	TCAGGTTGTACGGGATCAAAT<br>GTAGAGGTGCAAAGTTACCAGCC	GTTCGGCTGCCATTTGCAGA<br>TTAGAGCCTGGTCCTCCTTCTG
Tbx1	CTGTGGGACGAGTTCAATCAG	TTGTCATCTACGGGCACAAAG
Fgf10	TTTGGTGTCTTCGTTCCCTGT	TAGCTCCGCACATGCCTTC
Mef2c	ATCCCGATGCAGACGATTCAG	AACAGCACACAATCTTTGCCT

Tbx20	CGAGCAGCTCCTCAAACAGA	CCGTGGCTGGTACTTATGCA
Hand2	GAGAACCCCTACTTCCACGG	GACAGGGCCATACTGTAGTCG
Nkx2.5	GCTTCAAGCAACAGCGGTAC	ACTTGTAGCGACGGTTCTGG
Myl2	ATCGACAAGAATGACCTAAGGGA	ATTTTTCACGTTCACTCGTCCT
Cited2	TTCAAGTTGGCTGTCCCC	ACTGACGACATTCCACACCC

Supplementary References

1 de Soysa, T. Y. *et al.* Single-cell analysis of cardiogenesis reveals basis for organ-level developmental defects. *Nature* **572**, 120-124, doi:10.1038/s41586-019-1414-x (2019).
