# Supplementary material for: Single-cell transcriptomic profiling unveils dysregulation of cardiac progenitor cells and cardiomyocytes in a mouse model of maternal hyperglycemia
Source: Commun Biol. 2022 Aug 15;5:820. doi: 10.1038/s42003-022-03779-x (PMC9378651; doi:10.1038/s42003-022-03779-x)
Supplement: Supplementary file 3 — Description of Additional Supplementary Files [file 42003_2022_3779_MOESM3_ESM.pdf]

Description of Additional Supplementary Files

File name: Supplementary Data 1. List of DEGs in CNTRL vs. matHG-exposed E9.5 hearts
Description: Differentially expressed genes in control (CNTRL) vs. maternal hyperglycemia (matHG) exposed E9.5 hearts

File name: Supplementary Data 2. List of GO terms associated with DEGs in CNTRL vs. matHG-exposed E9.5 hearts
Description: Gene Ontology terms associated with differentially expressed genes in control (CNTRL) vs. maternal hyperglycemia (matHG) exposed E9.5 hearts

File name: Supplementary Data 3. List of DEGs in CNTRL vs. matHG-exposed E11.5 hearts
Description: Differentially expressed genes in control (CNTRL) vs. maternal hyperglycemia (matHG) exposed E11.5 hearts

File name: Supplementary Data 4. List of GO terms associated with DEGs in CNTRL vs. matHG-exposed E11.5 hearts
Description: Gene Ontology terms associated with differentially expressed genes in control (CNTRL) vs. maternal hyperglycemia (matHG) exposed E11.5 hearts

File name: Supplementary Data 5. List of DEGs in CNTRL vs. matHG exposed E9.5 MP-CM subclusters
Description: Differentially expressed genes in control (CNTRL) vs. maternal hyperglycemia (matHG) exposed E9.5 MP-CM subclusters

File name: Supplementary Data 6. List of DEGs in CNTRL vs. matHG-exposed E11.5 MP-CM subclusters
Description: Differentially expressed genes in control (CNTRL) vs. maternal hyperglycemia (matHG) exposed E11.5 MP-CM subclusters

File name: Supplementary Data 7. List of GO-terms in E9.5 and E11.5 MP-CM subclusters
Description: Gene Ontology terms associated with differentially expressed genes in E9.5 and E11.5 MP-CM subclusters

File name: Supplementary Data 8 E9.5 DEG analysis of MP-CM monocle states
Description: Differentially expressed genes in control (CNTRL) vs. maternal hyperglycemia (matHG) exposed E9.5 MP-CM pseudotime states

File name: Supplementary Data 9. E11.5 DEG analysis of MP-CM monocle states
Description: Differentially expressed genes in control (CNTRL) vs. maternal hyperglycemia (matHG) exposed E11.5 MP-CM pseudotime states

Description of FigShare Data
MatHG_E11.5: https://figshare.com/s/84ae58a8c28ff16cda0a
MatHG_E9.5: https://figshare.com/s/b31ea125f0aff94257f0
Control_E11.5: https://figshare.com/s/51ffd141bb0ed25fd163
Control_E9.5: https://figshare.com/s/deb29b59c0a9a271ce07
Source Data for Main Figures in the paper:

File name: Supplementary Data 10
Description: The source data behind Fig. 1e in the paper

File name: Supplementary Data 11
Description: The source data behind Fig. 2c in the paper

File name: Supplementary Data 12
Description: The source data behind Fig. 3b in the paper

File name: Supplementary Data 13
Description: The source data behind Fig. 3d in the paper

File name: Supplementary Data 14
Description: The source data behind Fig. 4c in the paper

File name: Supplementary Data 15
Description: The source data behind Fig. 4p in the paper

File name: Supplementary Data 16
Description: The source data behind Fig. 5i in the paper

File name: Supplementary Data 17
Description: The source data behind Fig. 5j in the paper

File name: Supplementary Data 18
Description: The source data behind Fig. 5p in the paper

File name: Supplementary Data 19
Description: The source data behind Fig. 7c in the paper

File name: Supplementary Data 20
Description: The source data behind Fig. 7f in the paper

File name: Supplementary Data 21
Description: The source data behind Fig. 7k in the paper
